# Catalytic Performance and Sulfur Dioxide Resistance of One-Pot Synthesized Fe-MCM-22 in Selective Catalytic Reduction of Nitrogen Oxides with Ammonia (NH_3_-SCR)—The Effect of Iron Content

**DOI:** 10.3390/ijms231810754

**Published:** 2022-09-15

**Authors:** Agnieszka Szymaszek-Wawryca, Urbano Díaz, Dorota Duraczyńska, Konrad Świerczek, Bogdan Samojeden, Monika Motak

**Affiliations:** 1 Faculty of Energy and Fuels, AGH University of Science and Technology, al. Adama Mickiewicza 30, 30-059 Krakow, Poland; 2 Instituto de Tecnología Química, UPV-CSIC, Universitat Politècnica de València-Consejo Superior de Investigaciones Científicas, Avenida de los Naranjos, s/n, 46022 Valencia, Spain; 3 Jerzy Haber Institute of Catalysis and Surface Chemistry, Polish Academy of Sciences, ul. Niezapominajek 8, 30-239 Krakow, Poland

**Keywords:** DeNO_x_, MCM-22, layered MWW zeolites, iron catalysts, SO_2_ poisoning

## Abstract

The catalytic performance of Fe-catalysts in selective catalytic reduction of nitrogen oxides with ammonia (NH_3_-SCR) strongly depends on the nature of iron sites. Therefore, we aimed to prepare and investigate the catalytic potential of Fe-MCM-22 with various Si/Fe molar ratios in NH_3_-SCR. The samples were prepared by the one-pot synthesis method to provide high dispersion of iron and reduce the number of synthesis steps. We have found that the sample with the lowest concentration of Fe exhibited the highest catalytic activity of ca. 100% at 175 °C, due to the abundance of well-dispersed isolated iron species. The decrease of Si/Fe limited the formation of microporous structure and resulted in partial amorphization, formation of iron oxide clusters, and emission of N_2_O during the catalytic reaction. However, an optimal concentration of Fe_x_O_y_ oligomers contributed to the decomposition of nitrous oxide within 250–400 °C. Moreover, the acidic character of the catalysts was not a key factor determining the high conversion of NO. Additionally, we conducted NH_3_-SCR catalytic tests over the samples after poisoning with sulfur dioxide (SO_2_). We observed that SO_2_ affected the catalytic performance mainly in the low-temperature region, due to the deposition of thermally unstable ammonium sulfates.

## 1. Introduction

The increasing emission of gaseous pollution is one of the major problems of European and international climate policy. However, on the industrial level, it is hard to give up relatively cheap energy sources, such as lignite or hard coal. Gases that play a critical role in atmospheric chemistry are nitrogen oxides (NO_x_). It is mainly due to their harmful influence on the environment: formation of photochemical smog and acid rain, or depletion of the ozone layer [1,2]. Selective catalytic reduction with ammonia or urea (NH_3_-SCR) is one of the dominant methods to remove NO_x_ produced by stationary emission sources [3,4]. This catalytic process is based on the reaction between nitrogen oxide and ammonia in the presence of oxygen and it yields molecular nitrogen and water vapor. The technology was developed in the 1970s in Japan and has become widespread around the world [5,6]. In recent years, the most common commercial catalytic system of NH_3_-SCR is V_2_O_5_-TiO_2_ promoted with MoO_3_ or WO_3_ [7,8]. In fact, the catalyst exhibits satisfactory performance in the temperature range of 300–400 °C [9], however, it is not free from some significant drawbacks [10,11]. The narrow temperature window forces the placement of the electrostatic precipitator and desulfurization unit downstream of the NH_3_-SCR installation in power plants. In this position, the exhausts reach sufficient temperature to activate the catalyst. Nonetheless, the material can be deactivated by heavy and alkali metals [12,13]. Alternatively, the commercial system can be placed in the so-called tail-end position, where the gases need to be re-heated. Such a procedure limits the problems of rapid deactivation; however, it demands higher energy consumption. Another disadvantage of the vanadium-based system is the activity in the oxidation of SO_2_ to SO_3_ [14,15,16]. The product interacts with NH_3_ below 300 °C, resulting in the formation of ammonium bisulfates (NH_4_HSO_4_) and ammonium sulfates ((NH_4_)_2_SO_4_), which cause pressure drops in the catalytic reactor [17,18]. Additionally, sulfur dioxide permanently deactivates metal oxide catalysts by the generation of thermally stable metal-sulfate deposits on redox centers [19,20]. Therefore, one of the most challenging issues in the design of effective SCR catalysts is the resistance to the harmful influence of SO_2_. 

In recent years, many research studies have focused on the application of supported metal oxides [2,21], natural aluminosilicates modified with transition metals [22,23], modified activated carbon [24,25], or waste materials [26,27] as NH_3_-SCR catalysts. Apart from that, a bunch of catalytic systems based on natural or synthetic zeolites have been examined [28,29,30,31]. The group of layered zeolites belonging to the MWW (Mobile tWenty tWo) family are among the most attractive as novel NH_3_-SCR catalysts. The unique structure and pore system of these aluminosilicates enables creation of the opened structure with easily accessible active centers [32]. One of the representatives of this group, MCM-22 synthesized for the first time in the 1990s, has been reported to be a highly active catalyst for many organic reactions [33,34,35,36]. This 2D-structured material possesses an unusual pore system accessible through 10-ring openings. In practice, it is a combination of two-dimensional sinusoidal channels that maintain a medium 10-ring diameter and large supercages with 12-ring cavities, with inner diameter and length of 7.1 Å and 18.2 Å, respectively [33,37]. In contrast to other zeolites, such as SSZ-13, MCM-22 can be prepared in a wider Si/Al range and with much lower cost [38,39]. The unusual features of MCM-22 were a premise to open a new path in the research over zeolite-supported DeNO_x_ catalysts. Palomares et al. [40] analyzed the potential of Co-exchanged MCM-22 in SCR with propane. It was found that medium size pores and the absence of large cavities of the material provide satisfactory catalytic conversion of NO. Rutkowska et al. [41] compared the catalytic performance of MCM-22, MCM-36 and ITQ-2 modified with iron or copper by ion-exchange method in SCR with ammonia. The authors claimed that among the tested materials, MCM-22 shows the highest catalytic potential in NO reduction by NH_3_. Additionally, the performance of the MWW zeolites was strongly correlated with their acidity, surface parameters, and the content and distribution of the active phase. Chen et al. [38] prepared a series of Cu-MCM-22 catalysts with various metal loadings using impregnation procedure. The obtained results showed that when the loading of copper exceeded 4 wt.%, the catalyst suffered from pore blockage by the aggregated metal oxide species. Alternatively, Chen et al. [42] tested MCM-22 modified with iron by various methods in SCR with ammonia. According to the authors, high conversion of NO over Fe-functionalized zeolite can be obtained only for the material that contains a significant amount of isolated metal species. It was found that so-called one-pot synthesis provides abundance of these moieties.

Several studies have suggested that modified zeolites exhibit moderately good resistance to the poisoning with SO_2_. Auvray et al. [43] compared the behavior of Cu-BEA and Cu-SZZ-13 in NH_3_-SCR in the presence of sulfur oxides. Their findings confirmed that the hindering influence of SO_2_ was strongly correlated with the size of the pores. The results were in line with that obtained by Hammershøi and co-workers [44], who confirmed that the determining factor for SO_2_ resistance was related to the zeolite structure. In general, the great majority of studies on SO_2_-poisoning of SCR catalysts is based on Cu-zeolites and only few authors have investigated the resistance of Fe-zeolites. Moreover, the catalytic performance of modified layered zeolites of MWW family poisoned with SO_2_ has not been yet examined.

According to the above-mentioned, the aim of this work was to broaden our knowledge of the application of Fe-modified MCM-22 in SCR process. With the intention of achieving the highest dispersion of iron within the zeolitic support, and preventing the deposition of bulk metal oxides in the pores, we used the one-pot method to introduce iron. In order to find the most appropriate content of the active phase, we applied various concentrations of Fe precursor in the synthesis slurry. The reference catalyst was prepared by post-synthesis ion-exchange procedure. To identify the catalytic performance and SO_2_ resistance of the synthesized materials, we conducted NH_3_-SCR catalytic tests using the model gas mixture over fresh and SO_2_-poisoned samples.

## 2. Results

### 2.1. Catalytic Performance of the Fresh Catalysts

**Figure 1:**
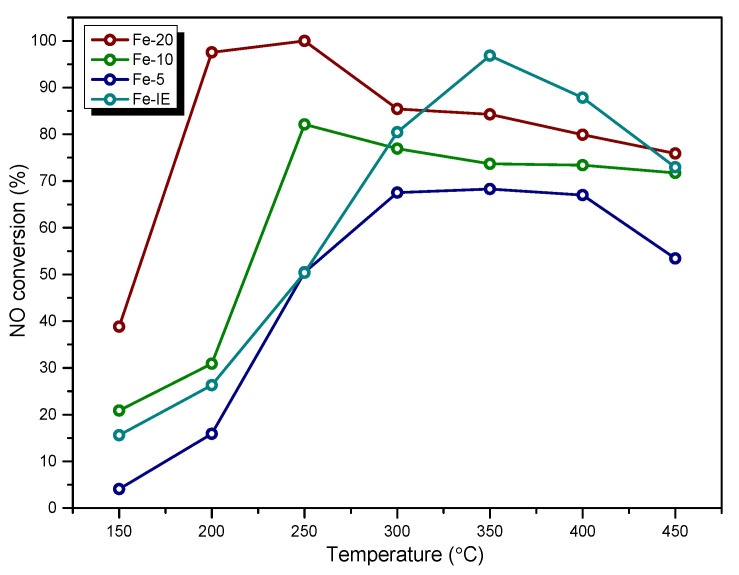
NO conversion obtained for the fresh catalysts.

NO conversion obtained for the fresh catalysts is presented in Figure 1. It can be noticed that Fe-20 exhibited the highest catalytic activity among all of the one-pot synthesized materials. The temperature of 50% NO conversion (*T_50_*) for the catalyst was around 155 °C, while at 175 °C almost 100% of NO was eliminated. On the other hand, Fe-10 and Fe-5 showed significantly lower catalytic activity. In general, NO conversion of the materials in the whole temperature range can be ordered as: Fe-5 < Fe-10 < Fe-20. Therefore, the high iron content in the zeolite did not determine satisfactory activity of the catalysts. Additionally, *T*_50_ of Fe-10 and Fe-5 were 220 and 250 °C, respectively. Thus, the increased amount of Fe in the catalysts declined their low-temperature SCR activity. Interestingly, in the case of Fe-20 and Fe-10, NO conversion remained stable within 300–450 °C. Similar result can be observed for Fe-5, however, the activity of this sample gradually decreased between 400 and 450 °C. Additionally, below 300 °C, one-pot synthesized Fe-catalysts showed considerably higher NO conversion than Fe-IE. The reference sample prepared by ion-exchange exhibited catalytic activity characteristic for the materials with iron introduced by the post-synthesis methods. *T_50_* of the sample oscillated around 250 °C and was almost as high as that of Fe-5. Therefore, one-pot synthesis contributed to low-temperature activity of the investigated Fe-zeolite catalysts.

**Figure 2:**
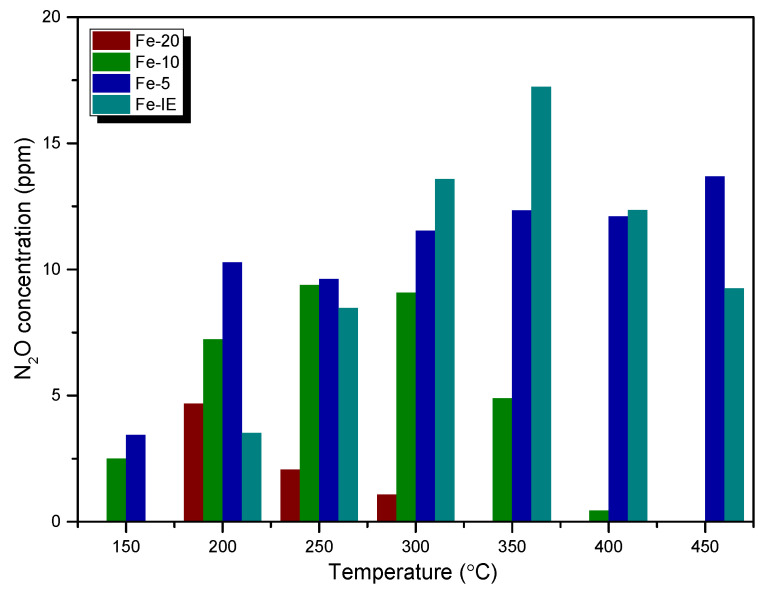
N_2_O concentration during the catalytic tests performed over the fresh catalysts.

The concentration of N_2_O (a common by-product of NH_3_-SCR) at the reactor outlet is another important factor, measured during the catalytic reaction. It can be noticed from Figure 2 that the emission of N_2_O did not exceed 20 ppm for any of the catalysts. However, for one-pot synthesized samples it increased linearly with Fe loading and the lowest N_2_O yield was obtained for Fe-20. The emission of the considered by-product for this sample was detected only within 200–300 °C, probably as the result of non-selective reactions occurring during decomposition of NH_4_NO_3_ below 260 °C [45]. N_2_O production decreased with the increasing temperature for Fe-10 as well, however, it was observed between 250–400 °C. This effect can be explained by the possible decomposition of nitrous oxide over the Fe_x_O_y_ clusters, present in the material (see XPS and UV-vis results) [46]. On the contrary, the N_2_O yield for Fe-5 was maintained at a similar level throughout the examined temperature range and was the highest among the one-pot synthesized catalysts. Interestingly, for Fe-IE, the concentration of N_2_O was considerably higher than that of the other samples in the high-temperature range of SCR. This result can be related to the side reaction of ammonia oxidation on the active sites of the catalysts [47].

### 2.2. Catalytic Performance of the SO_2_-Poisoned Catalysts

**Figure 3:**
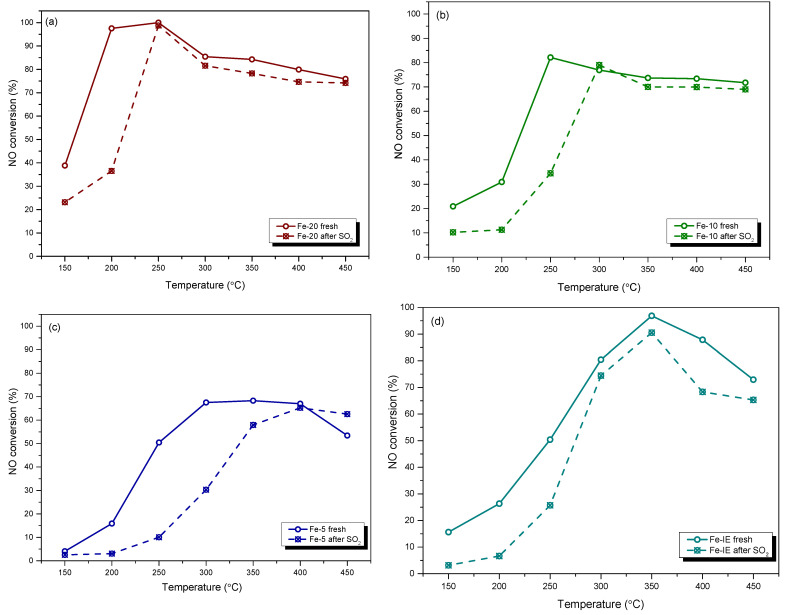
NO conversion obtained for the fresh and SO_2_-poisoned catalysts: (**a**) Fe-20; (**b**) Fe-10; (**c**) Fe-5; (**d**) Fe-IE.

Sulfur dioxide (SO_2_) belongs to the common pollutants present in industrial exhausts and causes significant problems in practical applications of the catalysts. Due to that, we performed NH_3_-SCR tests after poisoning of the materials with SO_2_. NO conversion obtained for the fresh and SO_2_-poisoned catalysts is demonstrated in Figure 3. One can notice that both Fe-20 and Fe-10 exhibited similar behavior after poisoning with SO_2_. *T_50_* of the catalysts increased from 155 to 205 °C and 205 to 255 °C, respectively. It suggests that introduction of the sulfate species inhibited adsorption of the reacting molecules and elevated the activation energy to initiate the catalytic reaction. This phenomenon can be assigned to the competitive adsorption of SO_2_ against NO and NH_3_ during the process [48]. However, in the case of Fe-20, the activity was almost completely restored only at 250 °C. Considering Fe-10, the most drastic poisoning effect was observed below 275 °C, while above this temperature, NO conversion was only slightly lower than that of the fresh sample. On the contrary, poisoning with SO_2_ dramatically lowered NO conversion obtained by Fe-5 within 150–400 °C and increased *T_50_* of the catalyst from 225 °C to ca. 330 °C. This effect can be explained by the formation of thermally-stable Fe_2_(SO_4_)_3_ deposits, which limited the access of the reacting molecules to the active centers. Such a different high-temperature behavior of Fe-5 comparing to Fe-20 and Fe-10 is related to the highest concentration of iron, thus, higher probability of Fe_2_(SO_4_)_3_ formation. However, it was observed that within 400–450 °C, NO conversion of the poisoned Fe-5 reached the same or even higher level than the fresh material. One possible explanation of this effect is that the formed iron sulphates blocked the active centers for non-desired reactions, such as ammonia oxidation. Last but not least, the catalytic activity of the poisoned Fe-IE was lower comparing to the fresh sample in the whole investigated temperature range. It was observed that *T_50_* of the material after poisoning was shifted from ca. 250 °C to 273 °C. Additionally, in contrast to the one-pot synthesized samples, NO conversion for the poisoned Fe-IE was not restored in the high-temperature range. Therefore, introduction of the active phase during the zeolite synthesis promoted the formation of iron sites with lower affinity to react with SO_2_ and form pore- and active-centers blocking deposits.

The concentration of nitrous oxide present in the aftertreatment gas for the fresh and SO_2_-poisoned catalysts is listed in Table 1. In general, the emission of N_2_O did not exceed 50 ppm for any of the studied materials. Thus, the result is satisfactory from the viewpoint of industrial applications. However, for all of the one-pot synthesized samples, the total production of N_2_O increased after poisoning. The most remarkable change was observed for Fe-5, while for Fe-20 and Fe-10 the effect was only negligible. Furthermore, the concentration of nitrous oxide detected for the poisoned Fe-IE was very close to that of the fresh material. Thus, the decreased NO conversion observed for this sample is not related to the generation of N_2_O via ammonia oxidation or other side-reactions of NH_3_-SCR.

### 2.3. Characterization of the Catalysts

#### 2.3.1. Chemical Composition and Crystal Structure

The amount of Si, Al, and Fe in the catalysts, as well as the real Si/Al molar ratios were measured by ICP-OES analysis. The results of the investigation are presented in Table 2. In the case of MCM-22, the real Si/Al molar ratio was slightly smaller comparing to the intended one, confirming that Al cations were more preferably built into the zeolitic framework comparing to Si cations. It was observed that in all of the analyzed samples Si content remained unchanged, regardless of the procedure of Fe introduction. Additionally, the increasing amount of iron introduced into the zeolite framework did not cause linear decrease of Al concentration. Thus, some part of the iron species did not replace Al^3+^, but was deposited in the extra framework positions, in the form of small Fe_x_O_y_ oligomers or larger aggregates of Fe_2_O_3_.

**Figure 4:**
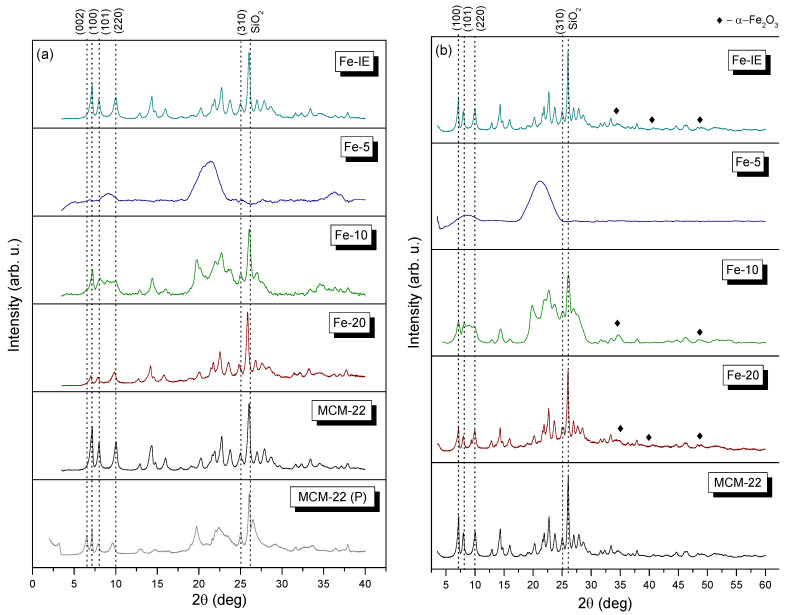
XRD patterns of the zeolites in the *2θ* range of (**a**) 3–40° and (**b**) 3–60°.

XRD analysis played a crucial role in the structural characterization of the catalysts. Additionally, the analysis enabled to determine the distance between the MWW layers of the selected samples. The XRD patterns obtained in a *2θ* range of 2–40° and 3–60° are presented in Figure 4a,b, respectively. Considering the pristine MCM-22, the most important reflections are grouped within *2θ* of 6–10° [49]. The relatively weak diffraction maximum detected for MCM-22 (P) at ca. 3.1° confirmed the formation of the layered structure [34], while the inter-layer (002) diffraction peak at *2θ* of around 6.5° reflects the regular separation achieved between the individual zeolitic layers. This reflection corresponds to the *d*-spacing of 1.3 nm, and therefore, the distance between two adjacent MWW layers, perpendicularly ordered to the *c* axis, can be estimated to be ca. 2.6 nm. Calcination of MCM-22 (P) yielded 3D zeolitic structure of MCM-22, confirmed by the overlap of (002) diffraction maximum with the intra-layer reflection (100). This result is assigned to the contraction of the zeolitic layers, caused by the condensation between the opposite external silanol groups, as the result of the removal of the organic template [46]. Additionally, the positions of the intra-layer (100) and inter-layer mixed (101) and (220) reflections did not noticeably change after calcination. As can be seen, the one-pot synthesized samples exhibited lower intensity of the characteristic diffraction maxima. Hence, introduction of iron into the synthesis pot restricted successful formation of MWW-type framework and decreased crystallinity of the materials. In the XRD pattern of Fe-20, all of the reflections characteristic for MWW structure can be distinguished. However, their intensity is significantly lower comparing to the reference sample. Furthermore, for Fe-10, the only MWW-characteristic diffraction maximum was that with *hkl* indices of (100). On the other hand, (101) and (220) maxima partly overlapped with each other. Surprisingly, any characteristic diffraction peaks were observed in the XRD pattern of Fe-5. Thus, the sample was found to be amorphous. Thus, it can be assumed that the excessive introduction of iron restricted the formation of layered structure, typical for MCM-22. Therefore, both Fe-10 and Fe-5 cannot be considered as the catalysts supported on zeolites belonging to MWW family. In summary, Fe content significantly affected the formation of the layered crystalline structure. The relative crystallinity (*RC*) calculated for the analyzed materials is presented in Table 2. The sum of the intensities of the diffraction peaks appearing at *2θ* of 14.3, 22.7, 23.7, and 26.0° for each catalyst was compared with that obtained for MCM-22, taken as the reference sample (all XRD measurements were conducted in the same conditions). The crystallinity of the materials increased following the order of Fe-5 < Fe-10 < Fe-20 < Fe-IE. In order to confirm the presence of iron species in the materials, XRD patterns of MCM-22 and the catalysts were recorded in the *2θ* range of 3–90°. The obtained results are depicted in Figure 4b. Basing on the shape of the patterns, it was found that iron is present in the catalyst in the form of well-dispersed cations or very small crystals of α-Fe_2_O_3_ (ICDD 89-599), confirmed by the diffraction maxima at *2θ* of 33.1, 40.1, or 49.5° [42].

#### 2.3.2. Structural and Surface Properties

**Figure 5:**
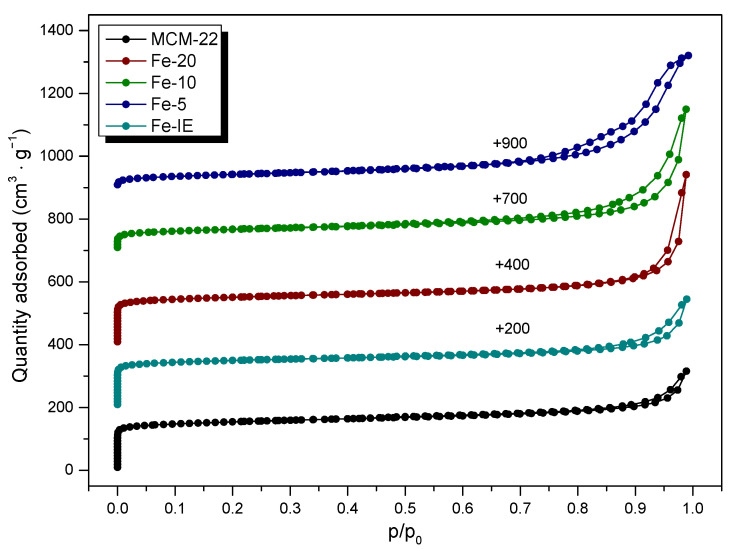
Nitrogen adsorption-desorption isotherms obtained for non-modified MCM-22 and the catalysts (for better visibility, the isotherms were shifted by the values given in the figure).

Nitrogen adsorption-desorption measurements were performed to investigate textural properties of the materials. The obtained results are presented in Table 3, while the isotherms are depicted in Figure 5. The adsorption-desorption branches exhibited by non-modified MCM-22 and the reference sample, Fe-IE were of type I (b), according to IUPAC classification, with a very narrow hysteresis loop of H4. The isotherms contained characteristic, sudden increase of the adsorbed N_2_ at very low *p*/*p*_0_, caused by the microporous structure of the materials [50]. Furthermore, the formation of hysteresis loop at *p*/*p*_0_ of ca. 1 indicated the presence of secondary slit-shaped mesopores or narrow macropores created by aggregated zeolite crystals [34,37,41,51]. Additionally, the uptake in N_2_ adsorption, observed in the range of 0.8 < *p*/*p*_0_ < 1.0 is related to the stacked holes between the layers [37]. It was observed that for one-pot synthesized Fe-catalysts, the shapes of the isotherms were determined by iron loading. For Fe-20, it remained almost unchanged in the *p*/*p*_0_ range of 0–0.9. The noticeable increase of N_2_ adsorption above this region resulted in the transformation of the hysteresis loop to H3, which points to the formation of non-rigid aggregates of plate-like particles [52]. The same effect was observed in the case of hysteresis loop of Fe-10. However, the sample showed significantly lower N_2_ adsorption capacity at very low values of *p/p*_0_, thus, lower contribution of microporosity, confirmed by the data presented in Table 3. The isotherm of Fe-5 was the most distinct among all of the materials. It can be classified as type IV(a), given normally by mesoporous materials, such as mesoporous molecular sieves. The hysteresis loop for the sample is a mixture of H3 and H5, associated with the presence of macropores and partially blocked mesopores, respectively [52]. The obtained effect probably results from the deposition of large aggregates of Fe_2_O_3_ present within pore openings of Fe-5. Importantly, the shape of the isotherms exhibited by Fe-10 and Fe-5 was additional confirmation that the catalysts cannot be qualified to the group of layered zeolites with MWW topology.

In can be found from the data collected in Table 3, that the pristine MCM-22 exhibited the specific surface area of 479 m^2^ g^−1^, which is typical value for this layered zeolite. The micropore and external surface area of the material correspond to that reported in the literature [38]. Introduction of iron by post-synthesis method decreased *S_BET_* of the zeolite by around 22%, while the area and the volume of micropores were around 28 and 32% lower than that of MCM-22, respectively. Therefore, some part of the introduced iron species was deposited in the micropores of MCM-22. Additionally, the loss of the *S_Ext_* value, observed for Fe-IE suggests that small aggregates of Fe_x_O_y_ were present on the external surface of the catalyst. The textural properties of the one-pot synthesized catalysts were strongly influenced by the Si/Fe molar ratio. In the case of Fe-20, the value of the specific surface area dropped only slightly, comparing to the non-modified zeolite and was the highest among the investigated catalysts. The value of the surface area of micropores of this catalyst is very close to that of MCM-22. Therefore, the majority of iron species in the sample are most likely located in the form of isolated Fe^3+^ cations, built into the zeolite framework. However, the decreased value of the external surface area and the micropore volume proved the presence of other kinds of iron sites, such as Fe_x_O_y_ aggregates, located within the pores and on the zeolite surface. Interestingly, all of the textural parameters of Fe-20 are superior to that of Fe-IE. Therefore, introduction of the controlled amount of the active phase during the zeolite synthesis is more beneficial for the structural features of the catalyst. One can notice that higher Fe loading in the materials led to the gradual decrease of the values of all of the structural parameters. In contrast to Fe-20, in the case of Fe-10, iron species blocked the great majority of the micropores. Furthermore, Fe-5 exhibited the lowest value of the specific surface area and micropores in the material were almost absent, which had a crucial impact on the catalytic performance of this material. 

**Figure 6:**
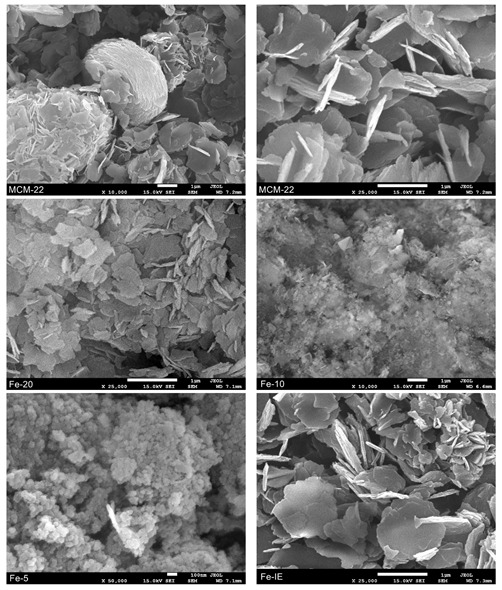
SEM images recorded for MCM-22 and Fe-catalysts.

The morphology of the non-modified MCM-22 and the catalysts was investigated by SEM microscopy. It can be noticed from Figure 6 and Figure S1 that the pure MCM-22 consists mainly of the sheet-like small particles. Additionally, the sheets formed relatively regular, donut-like aggregates, which is typical for the lamellar materials [34,53,54]. Furthermore, the reference sample, Fe-IE did not show any noticeable morphological differences, comparing to the support. In contrast, introduction of Fe into the synthesis pot caused some structural changes, observed in the recorded images. The particles of Fe-20 did not form any regular aggregates and occurred in the form of scattered, small plates. However, the particle diameter of this sample did not change significantly, comparing to that of MCM-22. Additionally, in line with the results of XRD, crystallinity of the materials decreased with the decreasing Si/Fe molar ratio. The characteristic MWW layers appeared only peripherally in the case of Fe-10, while Fe-5 was almost completely amorphous.

**Figure 7:**
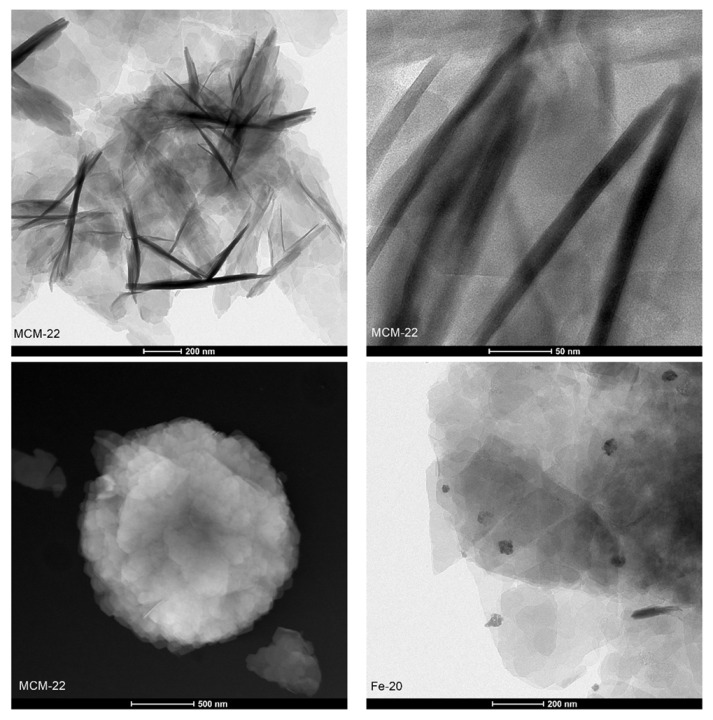

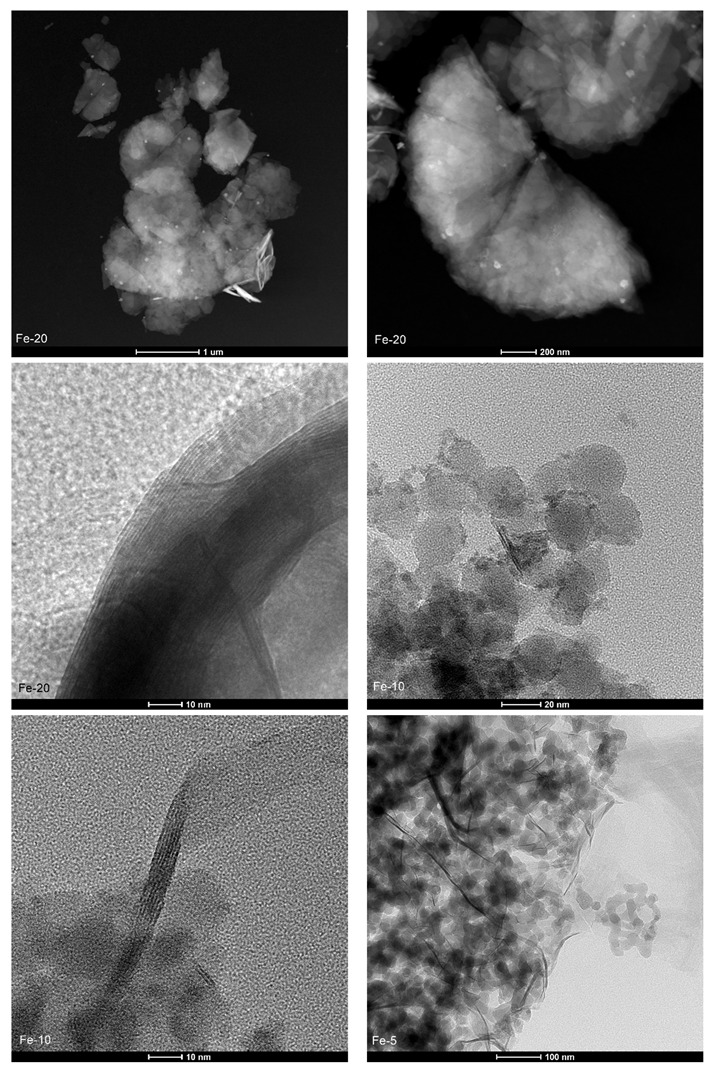

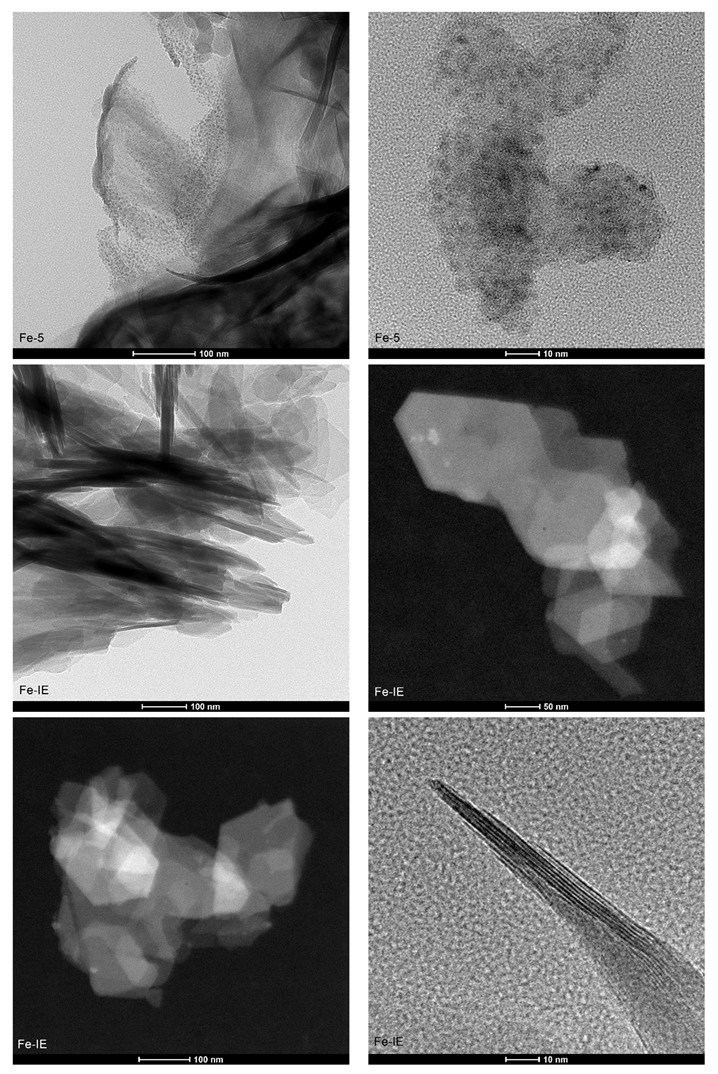
TEM images of the samples recorded in various modes.

Transmission electron microscopy (TEM) allowed to investigate directly the structure of the layered precursor and the catalysts on a nanoscopic scale. The micrographs recorded for the samples in bright-field (BF), high-angle annular dark-field (HAADF), and high resolution (HR) modes are presented in Figure 7. MCM-22, Fe-IE, and Fe-20 showed a plate-like morphology, due to the presence of ordered, piled MWW-type layers. The layers formed nanoscale crystallites, which can be distinguished into the regular arrays of the exposed 0.7 × 0.7 nm cups and the 10-ring channel system, when the individual layers are parallel to the microscope’s optical axis (see HR-TEM images). Additionally, although Fe-10 did not show the typical MWW crystal structure (according to XRD results), some individual layers can be distinguished in HR-TEM image of the material. However, as predicted, the structure of Fe-5 was not characteristic for the layered zeolites. 

Another benefit of TEM microscopy is that it enables analysis of the active phase introduced into the catalysts. Both Fe-20 and Fe-IE exhibited very well-dispersed iron species, visible in the HAADF images. Any agglomerated clusters were found while HR-TEM mode was applied, thus, iron in the materials had beneficial distribution for NH_3_-SCR. In contrast, Fe-10 and Fe-5 were abundant in bulky iron agglomerates, which contributed to the loss of crystallinity and restricted formation of the plate-like structure of the materials. The analysis of the samples by TEM microscopy in various modes is in line with the speciation of iron identified by the means of UV-vis spectroscopy.

**Figure 8:**
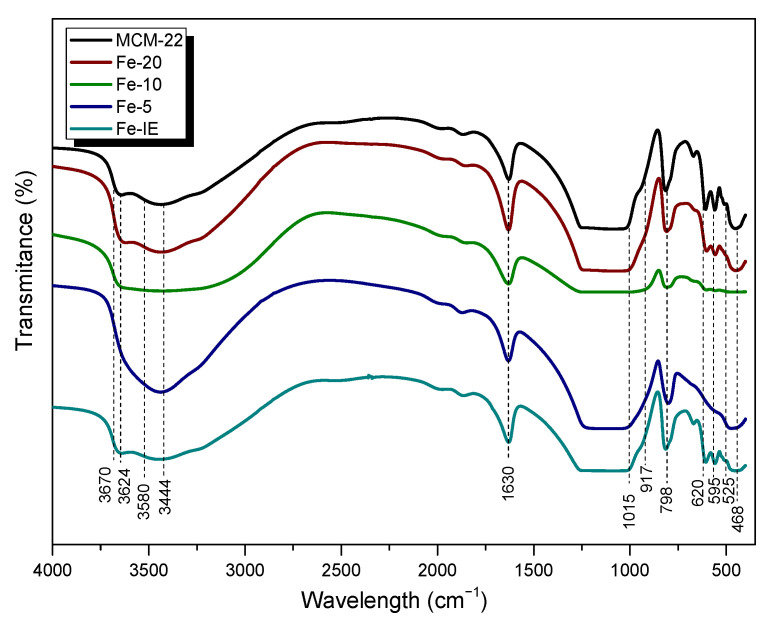
FT-IR spectra obtained for the samples.

The characteristic skeletal vibrations of the investigated materials were analyzed by FT-IR spectroscopy. The results depicted in Figure 8 showed that the spectra obtained for samples with Fe loadings of 0–6 wt.% are very similar. The peaks appearing in the wavelength range of 1300–400 cm^−1^ correspond to the vibrations of zeolitic framework [55]. The bands at 595 cm^−1^ and 525 cm^−1^, detected for MCM-22, Fe-20, and Fe-IE are assigned to the double-six-ring (D6R) of the MWW zeolite structure [51,56]. The absence of these peaks in the case of Fe-10 and Fe-5 points at some interruptions during the formation of the characteristic layered structure, caused by the excessive iron loading. Additionally, the presence of the band of asymmetric internal vibrations of T–O (where T = Si or Al) at 1015 cm^−1^ can be considered as the proof of the presence of Fe in all of the catalysts, except of Fe-5 [51,56].The absorption bands appearing for all of the samples at 798 and 917 cm^−1^ can be assigned to the stretching vibrations of SiO_4_^2^^−^ tetrahedra, while those detected at 525 and 468 cm^−1^ are be attributed to Al–O–Si and Si–O–Si bending vibrations, respectively. Another peak, at 620 cm^−1^ corresponded to the out-of-plane coupled vibrations of Al–O and Si–O [57]. Furthermore, the sharp peak at 1630 cm^−1^, appearing for all of the samples, confirmed that the materials contained physisorbed, structural water molecules [58]. It was observed that the shape of the spectra in the wavelength range of 4000–3000 cm^−1^, ascribed to the vibrations of OH groups, was strongly influenced by iron loading. All of the samples, except of Fe-5 exhibited characteristic peak at 3670 cm^−1^, assigned to Al-OH vibrations. The small band at 3624 cm^−1^ results from the Brönsted acidic sites Si(OH)Al, located in the supercages in 10-MR channels [59,60]. On the other hand, very weak band at 3580 cm^−1^ appeared due to the small fraction of Si(OH)Al species, located at the hexagonal prisms [61]. The peak at 3444 cm^−1^ is a confirmation of the hydrogen-oxygen bonds in OH groups [62].The different shape of the spectrum of Fe-5 in this wavelength region proved that the formation of zeolitic framework was significantly affected while Fe loading was ca. 15 wt.%.

**Figure 9:**
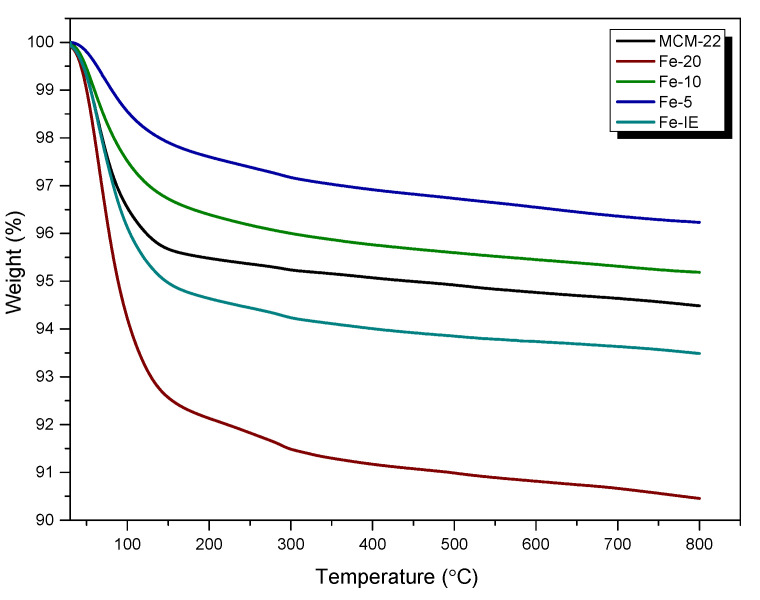
TGA results obtained for the samples.

Thermogravimetric analyses (TGA) of non-modified MCM-22 and the catalysts were carried out to determine their stability within 30–800 °C. The obtained TG curves are presented in Figure 9, and the calculated weight loss in the particular temperature regions are listed in Table 4. From the presented data, it can be assumed that the weight loss for any of the materials did not exceed 10% in the analyzed temperature region. Thus, the samples showed satisfactory thermal stability, which can be ordered as it follows: Fe-20 < Fe-IE < Fe-10 < Fe-5. Additionally, the absence of a noticeable weight loss of the samples above 300 °C proved that all of the organic precursors used during the preparation procedure were successfully removed. The decrease in weight, exhibited within 30–150 °C is related to the removal of moisture [53]. Interestingly, all of the catalysts prepared by one-pot synthesis showed similar behavior to the reference sample, Fe-IE. Additionally, the lowest weight loss observed for Fe-5 could be related to the deposition of bulky particles of Fe_2_O_3_, which hampered the migration of water molecules located in the internal structure of the zeolite. On the other hand, the noticeable difference between the stability of Fe-20 and other catalysts suggests higher concentration of the adsorbed water molecules, which could result from the adsorption of H_2_O on the isolated Fe^3+^ cations incorporated into the zeolitic framework.

#### 2.3.3. Speciation and Distribution of Iron

**Figure 10:**
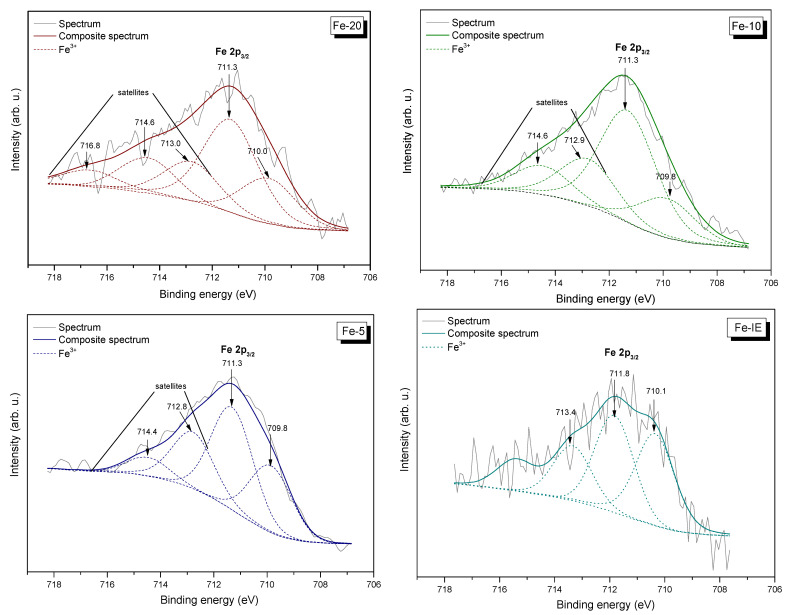
Fe 2p_3/2_ XP spectra of the catalysts.

Speciation and distribution of the chemical components on the catalysts’ surface was investigated by the means of XPS. The fragments of the obtained spectra, corresponding to the binding energy of Fe 2p_3/2_, along with the peak fitting are demonstrated in Figure 10. Furthermore, the fragments of the spectra assigned to the binding energies of O 1s, Si 2p, Al 2p, and C 1s are shown in Figures S2–S5, respectively. The relative areas of the peaks, given by the investigated components of the catalysts are listed in Table 5.

The results of XPS were analyzed (after appropriate subtraction of the linear background) using a Gaussian fitting procedure. It can be observed from Figure 10, that the peaks appearing at the lowest binding energy are located at around 709.8–710.1 eV (depending on the sample). This result confirmed the presence of Fe^3+^ species in the samples [63,64]. Furthermore, all of the materials exhibited satellites, sensitive for Fe oxidation state, located in the higher binding energy range. It must be added that the peaks ascribed to iron were broad (up to 6 eV), indicating that Fe was present in several different environments. The observed satellites are associated with the incorporation of iron into the MWW framework and/or its presence in the extra-framework positions, which changed the characteristics of the electric field induced by the zeolitic framework. Our results are in agreement with that obtained by Milanesio et al. [65], who modified MCM-22 with copper, which similarly to iron belongs to the group of d-electronic metals. For the Fe-IE sample (with the lowest Fe content, see Table 5), only one satellite was observed, probably due to uniform distribution of iron in the inner structure of the support [66]. On the other hand, Gurgul et al. [64] claimed that lower binding energies in the XP spectra are given by the aggregates of Fe_2_O_3_. Therefore, it can be assumed that Fe-10 and Fe-5 contained a higher concentration of extra-framework iron species, such as Fe_2_O_3_ particles. Additionally, as demonstrated in Table 5, the surface concentration of Fe^3+^ sites in the samples followed the order of Fe-IE < Fe-20 < Fe-10 < Fe-5.

As presented in Figure S2, the peak related to O 1s was fitted with three components. The first maximum, appearing at ca. 530.5 eV, proved the existence of lattice oxygen (denoted as O_β_) of surface metal oxides, such as O–Fe or O–Si. The second line, centered at 532.5 eV, confirmed the presence of defective oxygen in metal oxides (O–Fe, O–Si). Furthermore, the third line, found at 534.2 eV, was assigned to the oxygen present in –OH groups and/or H_2_O molecules (indicated as O_γ_), which were probably adsorbed during the contact of the samples with atmospheric air. The Si 2p spectra, presented in Figure S3**,** showed one doublet structure (the doublet separation p_3/2_—p_1/2_ equals ca. 0.6 eV), with the main line 2 p_3/2_ centered at 103.0 eV, related to the tetrahedral Si (IV) [64]. Moreover, the Al 2p spectra, shown in Figure S4, can be fitted with the single line, centered at 74.4 eV and assigned to the presence of Al^3+^ in the zeolite framework. Additionally, it can be observed from Figure S5 that the C 1s spectra were fitted with three components: aliphatic carbon (284.8 eV), C–O groups (286.6 eV) and C=O and/or O–C=O groups, evidenced with the line centered at 289.3 eV [67]. All of the detected lines are typical for organic contamination present on the surface of air-handled samples.

**Figure 11:**
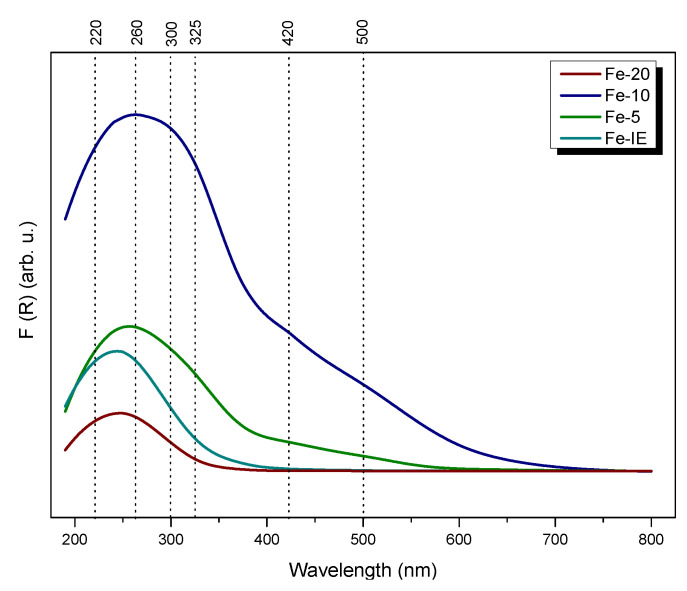
UV-vis spectra obtained for the catalysts.

Speciation and distribution of iron in the catalysts was characterized by UV-vis-DR spectroscopy. As shown in Figure 11, the form of iron in the samples was strongly influenced by its loading. All of the catalysts showed characteristic absorption bands in range of 200–600 nm and the spectra can be divided into four regions, ascribed to different iron species. The oxygen-to-metal charge-transfer bands below 300 nm, present for all of the materials, are assigned to the isolated, monomeric Fe^3+^ cations in various coordination [37,42,46]. The particular position of the specific bands depends on the number of ligands. Isomorphously incorporated, tetrahedrally coordinated iron cations in Fe-silicate were confirmed by the bands at ca. 220 nm [68]. Furthermore, the peak at 260 nm suggested the presence of isolated octahedral iron cations in the samples [69]. The absorption region located around 325 nm confirmed the existence of small oligomeric Fe_x_O_y_ species, while the band at 500 nm corresponded to bulky Fe_2_O_3_ particles, respectively [42,70]. What is important, the bands above 300 nm were absent for Fe-20. Thus, the sample was the most abundant in well-dispersed isolated Fe^3+^ sites. Basing on this result, it can be concluded that higher Si/Fe molar ratio provided better control of iron distribution in the catalysts. What is more, comparing to Fe-20, the reference sample, Fe-IE showed more intense absorption bands around 300 nm and above this value, which evidenced formation of polynuclear iron species in the material. Therefore, one-pot synthesis was more beneficial for the incorporation of the isolated Fe^3+^, comparing to ion-exchange method. As confirmed by TEM microscopy and XPS results, Fe-10 and Fe-5 contained high amount of polynuclear Fe_x_O_y_ and Fe_2_O_3_ clusters. This effect is most clearly visible for Fe-5, which showed the most intense absorption band among all of the materials, related to the highest Fe loading. Additionally, despite both Fe-10 and Fe-5 exhibited absorption bands below 400 nm, the possibly formed Fe active centers could be partially blocked by catalytically-passive Fe_2_O_3_ particles. The obtained results suggested that iron loading in the one-pot synthesized samples directly influenced the diversity of its species and as a consequence, catalytic performance in NH_3_-SCR. According to Brandenberger et al. [71], the behavior of iron-modified zeolites in SCR is strongly determined by the form of Fe in the zeolitic framework. The studies proved that the monomeric Fe^3+^ sites had a major contribution into the SCR activity below 300 °C. The effect is caused by the lowest activation energy of around 36 kJ mol^−1^. Thus, the application of the one-pot synthesis method and control of the amount of the active phase can increase its activity in low-temperature NH_3_-SCR.

#### 2.3.4. Surface Acidity

**Figure 12:**
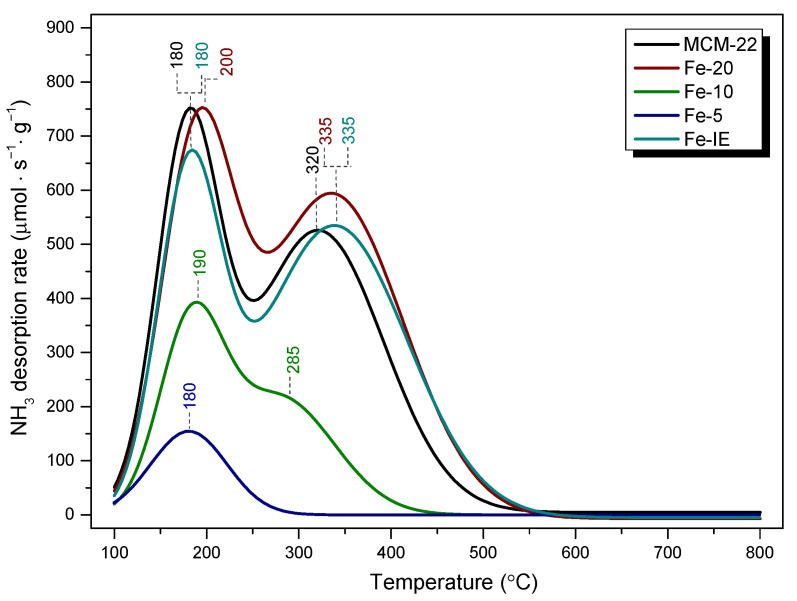
NH_3_-TPD profiles obtained for MCM-22 and the catalysts within 100–800 °C.

The adsorption and activation of ammonia on the catalyst surface is one of the crucial steps of NH_3_-SCR [72,73]. Therefore, acidic character of the materials was investigated by temperature-programmed desorption of ammonia (NH_3_-TPD). According to Kapustin and co-workers [74], determination of the adsorption heat of ammonia gives a valuable information on the general distribution of the weak and strong acid centers. However, although this method has been widely used for the determination of Brönsted acid sites in zeolites, one should note that strong adsorption of ammonia occurs also on Lewis acid sites [75]. NH_3_-TPD profiles obtained for in the temperature region of 100–800 °C are presented in Figure 12. The characteristic desorption peaks were deconvoluted into Gaussian curves (see Figures S6–S10), in order to facilitate the assignment to the corresponding temperatures. The quantitative outcomes of the analysis are listed in Table 6.

As presented in Figure 12, the investigated materials exhibited characteristic peaks within 180–200 °C and 300–335 °C, ascribed to the desorption of ammonia from weak (low temperature peaks, LTP) and medium or strong acid sites (high temperature peaks, HTP), respectively [34]. In general, the acidic character of the materials increased, following the order of Fe-5 < Fe-10 < Fe-IE < MCM-22 < Fe-20. For the pristine MCM-22, the desorption peaks are located at 180 °C (LTP) and 320 °C (HTP). The LTP could appear due to the NH_4_^+^ ion, weakly bonded to Brönsted acid sites, or due to the presence of framework and extra framework aluminum species in the zeolite. On the other hand, HTP can be assigned to the desorption of ammonium ions from the strong Brönsted sites, or NH_3_ molecules coordinated to Lewis acid sites [75,76]. According to the literature studies, modification with iron improves the medium and strong acidity of the zeolites by the introduction of the extra framework Fe^3+^ species, thus, strong Lewis acid centers [77]. Such effect could be observed only in the case of Fe-20 and the reference sample, Fe-IE. Additionally, LTP of Fe-20 was centered at 200 °C, hence, the weak acid sites of the sample bonded ammonia stronger than MCM-22 and Fe-IE. Furthermore, HTPs of both Fe-20 and Fe-IE were centered at 335 °C. Therefore, the samples contained slightly stronger acid sites of high strength than MCM-22. One can note that in contrast to Fe-IE, the intensity of LTP of Fe-20 is almost equal to that of MCM-22. These results differ slightly from those reported by Ates [75], who postulated that the density of the Brönsted acid centers decreases as the result of the exchange of Fe^3+^ with NH_4_^+^ cations. However, the author considered only post-synthesis modification of zeolites (ion-exchange). Therefore, our results revealed that the controlled introduction of the active phase into the synthesis pot limited the reduction of the Brönsted sites, caused by the modification with Fe. Additionally, according to Ryu et al. [78], the newly formed weak acid sites appear due to the creation of Lewis acid centers. In the case of Fe-10, LTP was shifted to 190 °C, however, the HTP, centered at 285 °C was hardly observed, since it partially overlapped with LTP. For Fe-5, LTP did not change its position, comparing to MCM-22, nevertheless, HTP completely disappeared. The observed phenomena can be explained by the fact that the majority of iron species in Fe-10 and Fe-5 was present in a form of clustered aggregates, which significantly lowered NH_3_ adsorption capacity of the materials. Additionally, since Fe-10 and Fe-5 did not show the characteristic frameworks of the layered zeolites (see XRD), their structures were much poorer in the acidic centers, characteristic for highly crystalline MWW materials.

## 3. Discussion

As presented in the Results section, the procedure of iron introduction and its content had a significant impact on physicochemical and catalytic features of the analyzed Fe-catalysts. Importantly, the results revealed that the formation of the characteristic structure of MWW zeolites was drastically disturbed while Si/Fe molar ratio was lower than 10. The decrease in specific surface area and disappearance of microporosity resulted from the deposition of bulky aggregates of iron oxide, detected for the materials with Si/Fe < 20. This conclusion is supported by XPS and UV-vis analyses, which indicated that the surfaces of Fe-10 and Fe-5 were much more abundant in Fe_2_O_3_ particles than Fe-20 and the reference sample, Fe-IE. Additionally, the results of TG studies indicated that evacuation of H_2_O molecules from the structure was somehow hindered for the materials with Si/Fe < 20 and the reference post-synthesized catalyst. This effect was likely caused by the presence of iron oxide particles, which hampered free diffusion of water from the pores. Moreover, the recorded SEM and TEM images confirmed that non-modified MCM-22 and Fe-catalysts exhibited different morphologies. Fe-5, with the highest loading of iron, was found to be almost completely amorphous, which was in line with XRD pattern obtained for this sample. On the other hand, despite some noticeable changes in the textural parameters of Fe-20 and Fe-10, the materials preserved crystalline structure. Therefore, we concluded that introduction of Fe to the synthesis pot of MCM-22, up to a certain level, did not disturb the formation of the zeolitic framework, as estimated by the calculation of relative crystallinity. What is important, the results of N_2_ sorption studies indicated that the extensive amount of iron introduced during the synthesis significantly affected formation of the microporous structure of the materials. Therefore, not only unsatisfactory distribution of iron, but also structural parameters contributed to lower catalytic activity of Fe-10 and Fe-5, comparing to Fe-20 and Fe-IE.

**Figure 13:**
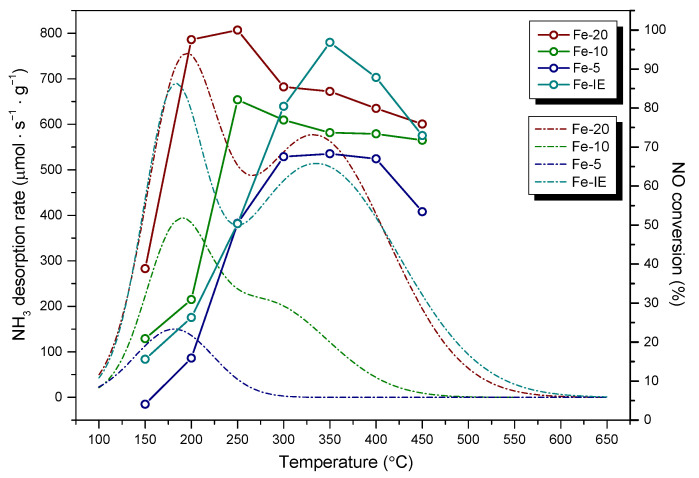
NH_3_-TPD profiles vs. NH_3_-SCR activity of the samples.

Apart from the structural features, the samples showed different acidic properties. It was found that the ability to adsorb ammonia increased with the decreasing content of iron in the zeolite. The effect was correlated with speciation of Fe, evidenced by UV-Vis spectroscopy. In our case, the highest amount of NH_3_ was adsorbed by Fe-20 and the material contained almost exclusively isolated iron cations in tetrahedral or octahedral coordination. Thus, the sites most actively interacted with ammonia molecules. What is more, it was observed that in comparison to Fe-IE, the density of acid sites of Fe-20 was not reduced as a consequence of the presence of iron, which typically occurs in the case of post-synthesized Fe-catalysts. The markedly higher concentration of the acid centers of Fe-20 can be explained by (1) the formation of Brönsted and Lewis centers in the form of aluminum species [76] and (2) abundance of the sample in isolated Fe^3+^ cations, which contribute to higher Lewis acidity [77]. Another important observation is that the density of acid sites in the samples decreased linearly with their specific surface area and micropore surface area. It can be noticed that concentration of acid centers is the highest for Fe-20 and Fe-IE, which exhibited well-developed texture. On the other hand, acidic character of Fe-10 and Fe-5 was visibly weaker. This effect can be explained by the presence of iron oxide clusters, which limited the access of NH_3_ molecules to the acidic adsorption centers in the catalysts. Additionally, insufficient development of pore structure could limit diffusion of ammonia molecules, which entered the samples. In contrast, Fe-20 and Fe-IE were abundant in the isolated Fe^3+^ cations, which not only acted as Lewis acid centers, but also did not block the pores of the catalysts. Our results correspond to that obtained by Andonova et al. [79] for Fe-SAPO-34. The authors observed that pore-blocking Fe_x_O_y_ clusters and more aggregated iron oxide species cause the loss of NH_3_ storage capacity. What is more, the correlation of the value of specific surface area and acid sites density in the cited research exhibited similar trend to that obtained within our work. Thus, decreased specific surface area, caused by the migration of Fe^3+^ cations and formation of pore-blocking particles, contributed to lower ammonia storage capacity. In order to investigate the correlation between surface acidity and catalytic activity of the materials, the results of NH_3_-TPD and NH_3_-SCR were presented in Figure 13. The noticeable correlation between the abundance of acid sites and high NO conversion was observed only in the case of Fe-20, since the highest activity of the sample corresponded to the maxima of NH_3_ desorption peaks in its TPD profile. Additionally, even though the density of acid sites of medium or strong strength was considerably lower or absent for the samples with Si/Fe < 20, the catalysts showed satisfactory high-temperature activity. This result is likely caused by the fact that Fe-20 contained not only isomorphously substituted Fe^3+^ species, which give rise to low-temperature activity, but also acid sites delivered by Al^3+^ and Al-OH sites, corresponding to Lewis and Brönsted acid centers, respectively. In the case of Fe-10 and Fe-5, these sites were absent or partially blocked by the clustered aggregates of Fe_2_O_3_. However, one can notice that despite Fe-10 contained very limited number of strong acid sites, it was relatively active in the high-temperature region of NH_3_-SCR. Similar phenomenon was noticed for Fe-5, which did not contain any strong acid centers. Additionally, catalytic activity of these two samples was relatively low in the low-temperature region. Considering Fe-IE, the highest NO reduction was reached above 250 °C, thus, not in the temperature range of the highest acidity. Hence, acidic features of both the one-pot synthesized materials and the reference sample were not the key factor determining their catalytic activity. Our findings support the postulate of Brandenberger and co-workers [80], who declared that especially Brönsted acidity is not required for the adsorption and activation of ammonia during NH_3_-SCR. Therefore, it can be assumed that the activity of one-pot synthesized materials with Si/Fe < 20 and Fe-IE was determined rather by the presence of polynuclear Fe^3+^ species or bulky Fe_2_O_3_ particles, which is in agreement with findings reported in the literature [69,70]. However, these species also contribute to a side-reaction of NH_3_-SCR, such as NH_3_ oxidation and its undesired consumption [69,70]. As a result, the presence of iron sites of higher nuclearity decreased activity of Fe-5 and Fe-IE above 375 °C. Our findings also support Luo et al. [81] hypothesis that the rate determining step for the standard SCR reaction over Fe-zeolite catalysts is NO oxidation. Insufficient amount of iron sites adsorbing NO could lead to the adsorption of NH_3_ on the acidic sites and its successive, undesired oxidation, especially above 400 °C. Side reactions of NH_3_-SCR were also catalyzed by bulk particles of Fe_2_O_3_, which presence were clearly evidenced by TEM images recorded Fe-5.

Altogether, the highest activity exhibited by Fe-20 resulted from the abundance of monomeric iron species, which was confirmed by physicochemical characterizations. What is more, Brandenberger et al. [73] proved that monomeric and dimeric Fe^3+^ sites have the major contribution to NO conversion below 300 °C. This postulate is in agreement with the presented results, since no other iron species have been detected in Fe-20. Another important factor that could influence the best catalytic performance of this sample is the highest specific surface area, and well-developed pore structure, which provided unrestricted diffusion of the reacting gas mixture through the zeolite channels and adsorption of the reactants on the active sites. In contrast, the decreased activity of Fe-20 above 275 °C was due to the absence of other iron species, such as oligomers or uncoordinated iron sites in the outmost layer of the zeolite. According to Kumar et al. [69] those sites are more active in high temperature range of SCR (300–450 °C), however, may also catalyze side-reactions and decrease selectivity to N_2_. Furthermore, the highest concentration of isolated Fe^3+^ species in Fe-20 resulted in the lowest amount of N_2_O emitted during the reaction. Zhang et al. [82] carried out the experiment of N_2_O formation over Fe-zeolite catalysts in various reaction environments. The authors postulated that the most probable pathway of N_2_O generation can be described by Reactions (R1)–(R5):

4NO + 4NH_3_ + 3O_2_ → 4N_2_O + 6H_2_O (R1)

2NH_3_ + 2O_2_ → N_2_O + 3H_2_O (R2)

2NH_3_ + 4NO → 2N_2_ + N_2_O + 3H_2_O (R3)

3NO → N_2_O + NO_2_ (R4)

4NO → 2N_2_O + O_2_ (R5)

Another probable explanation of the formation of N_2_O during the reaction is the Langmuir-Hinshelwood mechanism, which assumes interaction of NO_x_ and ammonia, both adsorbed on the catalyst surface. Ammonium nitrate (NH_4_NO_3_) is formed in the reaction as a solid or liquid and decomposes to nitrous oxide and water below 260 °C, according to the Reaction (R6) [45]:

NH_4_NO_3_ → N_2_O + 2H_2_O (R6)

Taken together, the increased concentration of N_2_O observed for all of the investigated catalysts at around 200–300 °C was a consequence of the Langmuir-Hinshelwood (LH) mechanism. Additionally, it can be noticed that for Fe-20 and Fe-10, the concentration of nitrous oxide decreased with the increasing temperature. This phenomenon can be correlated with the findings reported by Delahay et al. [83] who postulated that N_2_O is be reduced by NH_3_ to nitrogen and water vapor, as described by reaction (R7):

2NH_3_ + 3N_2_O → 4N_2_+ 3H_2_O (R7)

Since the process takes place mainly on the isolated Fe^3+^ cations in a distorted tetrahedral environment, it could occur simultaneously with NO-SCR over Fe-20 and Fe-10. An alternative explanation of the decreasing amount of N_2_O can be found in the content and speciation of iron. Rutkowska and co-workers [46] investigated the catalytic activity of Fe-modified MCM-22 in N_2_O decomposition. In their research, the authors recognized oligomeric iron species as the key active centers, which catalyzed the reaction. Characterizations of Fe-10 showed that such moieties were present in the structure of the material. Thus, the sample could be potentially active in N_2_O decomposition around 400–450 °C, which was proved by its rapidly decreasing concentration in this temperature region. Additionally, lower emission of nitrous oxide is not related to side reactions of NH_3_-SCR, such as ammonia consumption, since NO conversion within 400–450 °C was maintained at a constant level. Interestingly, Fe-10 was the only sample, which exhibited such behavior in terms of N_2_O elimination during the catalytic reaction.

The results of catalytic activities of SO_2_-poisoned catalysts showed that the influence of SO_2_ can be considered in various aspects. Firstly, ammonium sulfates and ammonium bisulfates can be formed during the interaction of NH_3_ with the poisoned catalyst. In this case, the reaction rate is limited due to lower specific surface area, pore volume, and pore size. However, the ammonium sulfates limit the activity only up to around 280 °C, since above this value these species are thermally decomposed [84]. Secondly, interaction of SO_2_ with metallic active phase leads to the formation of metal sulfate salts and irreversible deactivation of the catalyst [85]. Basing on the obtained results, it can be concluded that decreased catalytic activity of Fe-catalysts was a consequence of the combination of these two phenomena. Nagaishi et al. [86] suggested that Fe_2_O_3_-(NH_4_)_2_SO_4_ decomposes gradually with the increasing temperature and the process starts at around 270 °C. This temperature is close to that of the increase of NO conversion for both Fe-20 and Fe-10. Therefore, such species are likely to be present on the catalyst surface and could be the reason for the limited NO conversion below this temperature. Moreover, Song et al. [87] provided a detailed investigation of the mechanism of thermal decomposition of ammonium sulfate catalyzed by iron oxide. According to the authors, decomposition of (NH_4_)_2_SO_4_ takes place stepwise, as described by reactions (R8)–(R11):

6(NH4)2SO4 + Fe2O3 → 2(NH4)3Fe(SO4)3 + 6NH3 ↑ + 3H2O ↑(R8)

4(NH_4_)_3_Fe(SO_4_)_3_ + Fe_2_O_3_ → 6NH_4_Fe(SO_4_)_2_ + 6NH_3_ ↑ + 3H_2_O ↑ (R9)

6NH_4_Fe(SO_4_)_2_ → 3Fe_2_(SO_4_)_3_ + 6NH_3_ ↑ + N_2_ ↑ + 6H_2_O ↑ + 3SO_2_ ↑ (R10)

2Fe_2_(SO_4_)_3_ → 2Fe_2_O_3_ + 6SO_2_ ↑ + 3O_2_ ↑ (R11)

Reactions (R8) and (R9) start at around 250 and within 285–340 °C, respectively. Both of them are attributed to the release of ammonia, water vapor, and generation of (NH_4_)_3_Fe(SO_4_)_3_ or NH_4_Fe(SO_4_)_2_. Subsequently, the oxidation-reduction reaction (R10) between 355–440 °C is initiated. In this step, the solid deposit of Fe_2_(SO_4_)_3_ is formed and at the same time NH_3_, N_2_, H_2_O are emitted. The last step, described by reaction (R11), begins within 470–650 °C, which is outside the considered temperature range of NH_3_-SCR [10,88]. Due to this, with high probability, no Fe_2_O_3_ or O_2_ molecules are produced under the conditions of the studied reaction. However, the results presented by the authors showed significant relationship between the presence of iron species in the analyzed MWW-based catalysts and their catalytic behavior after poisoning with SO_2_. Basing on the Reactions (R8)–(R10), it can be assumed that the minor decrease of NO conversion after poisoning was caused by the decomposition of ammonium sulfates, catalyzed by iron species. Thus, during SCR reaction over the poisoned samples, iron oxide could act not only as an active phase, but also it helped to maintain or restore the catalytic activity. Such assumption is adequate especially for Fe-10, which possessed considerable amount of oligomeric and aggregated iron species. This result is also confirmed by the small difference between the activity of the fresh and poisoned material in the range of 275–450 °C. In the case of SO_2_-poisoned Fe-5, *T*_50_ increased to around 315 °C. It can be assigned to the formation of deposits of iron sulfate (Fe_2_(SO_4_)_3_), which occupied active sites of the material. A somewhat remarkable observation made for that sample is that after poisoning, the conversion did not decrease above 400 °C, as it did for the fresh material. This phenomenon can be explained by the occupation of iron sites present on Fe-5 by SO_2_, thus, hampered NH_3_ adsorption and its undesirable oxidation.

What is important, catalytic activity exhibited by the poisoned catalysts supported the theory that below 200 °C NH_3_-SCR reaction follows Langmuir-Hinshelwood mechanism (L-H), while above this value, the process transforms into Eley-Rideal (E-R) mechanism. In the case of L-H, both NO and NH_3_ are adsorbed on the active sites of the catalyst [89]. The initial treatment with SO_2_ resulted in the competitive adsorption with NO in the temperature range of 150–250 °C. Thus, further interaction of nitrogen oxide with the surface was hindered. Additionally, ammonium sulfates and bisulfates limited adsorption capacity of the catalysts, since these species occupied active centers up to 280 °C. As a result, activity of the poisoned materials was affected mainly in low-temperature range. In contrast, one can observe that at higher temperature, when the reaction typically follows E-R mechanism, the catalytic activity of the poisoned samples was much closer to that exhibited by the fresh catalysts. According to the general agreement, E-R mechanism assumes the adsorption of ammonia on the catalyst surface and its direct reaction with NO from the gaseous phase [90]. Therefore, the newly formed SO_4_^2^^−^ centers, introduced during poisoning, could be the reason of partial restoration of the catalytic activity above 250–280 °C (depending on the catalyst). These findings are in agreement with the earlier studies over SO_2_-poisoned catalysts, including zeolites [91,92]. Importantly, our results indicated that after poisoning the catalysts formed higher amount of N_2_O during the reaction. Taking into consideration both the existence of E-R mechanism inducted by the presence of SO_4_^2^^−^ [93] and additional amount of ammonia formed via reactions (R8)–(R10), we assumed that N_2_O is mainly generated due to the oxidation of NH_3_.

## 4. Materials and Methods

### 4.1. Catalyst Preparation

#### 4.1.1. Synthesis of MCM-22 Zeolite

MCM-22 with Si/Al molar ratio of ca. 25 was obtained according to the procedure described by Corma et al. [94]. The molar composition of the synthesis gel was SiO_2_:0.02 Al_2_O_3_:0_._5 HMI:0.09 NaOH:45 H_2_O. The synthesis mixture was prepared using sodium hydroxide (NaOH) (Sharlab, Barcelona, Spain), sodium meta-aluminate (NaAlO_2_) (56% Al_2_O_3_, 37% Na_2_O) (Carlo Erba Reagents, Milan, Italy), silica (Aerosil 200, Evonik Rohm Gmbh, Essen, Germany), hexamethyleneimine (HMI, 98 wt.%) (Sigma Aldrich, Merck KGaA, Darmstadt, Germany), and deionized water. The detailed preparation procedure was as it follows: 0.375 g of NaOH and 0.375 g of NaAlO_3_ were dissolved in 81.71 g of deionized water and stirred for 15 min at room temperature. Afterwards, 6 g of silica were slowly added to the mixture, maintained under stirring. Finally, 4.96 g of HMI was introduced dropwise into the solution and the resulting gel was mixed for 2 h. Subsequently, the slurry was crystallized in a stainless-steel autoclave lined with teflon under rotation (60 rpm) at 150 °C for 7 days. The obtained solid product was filtered, washed to neutral pH, and dried at 100 °C overnight in air. Finally, the precursor, labeled as MCM-22 (P) was calcined at the following temperature ramps: 100 °C for 2 h, 150 °C for 2.5 h, 350 °C for 3 h, and 580 °C for 3 h. The calcined form of the zeolite was labeled as MCM-22.

#### 4.1.2. Synthesis of the Catalysts

One-pot synthesized Fe-MCM-22 catalysts with Si/Al of ca. 28 and various Si/Fe molar ratios were prepared basing on the reproduced methodology described by Chen et al. [42]. In order to obtained the samples, a mixture of silica sol (40.5 wt.% of SiO_2_) (Sigma Aldrich, Merck KGaA, Darmstadt, Germany) sodium hydroxide, sodium meta-aluminate (NaAlO_2_), iron nitrate (Fe(NO_3_)_3_ 9H_2_O) (Sigma Aldrich, Merck KGaA, Darmstadt, Germany), hexamethyleneimine (HMI, 98 wt.%), and deionized water was used. The molar composition of the synthesis gel was SiO_2_:0.017 Al_2_O_3_:0.05 Fe(NO_3_)_3_:0.5 HMI:0.4 NaOH:45 H_2_O for Si/Fe = ca. 20, SiO_2_:0.017 Al_2_O_3_:0.1 Fe(NO_3_)_3_:0.5 HMI:0.4 NaOH:45 H_2_O for Si/Fe = ca. 10, and SiO_2_:0.017 Al_2_O_3_:0.2 Fe(NO_3_)_3_:0.5 HMI:0.4 NaOH:45 H_2_O for Si/Fe = ca. 5, respectively. Typically, the preparation procedure involved dissolution of the appropriate amounts of NaOH, NaAlO_2_, and Fe(NO_3_)_3_ 9H_2_O in deionized water and stirring the mixture for 10 min at room temperature. Afterwards, silica sol was added dropwise to the mixture and the adequate amount of HMI was introduced. The synthesis gel was stirred vigorously for 2 h. Subsequently, it was crystallized in stainless-steel autoclave lined with teflon under rotation (60 rpm), at 150 °C for 7 days. The resulting solid material was filtered, washed with distilled water to neutral pH, and dried at 100 °C overnight in air. Calcination of the catalyst was carried out at 560 °C for 10 h in air. The final calcination temperature was reached with the heating rate of 3 °C min^−1^. The obtained samples were labeled as Fe-20 Fe-10, and Fe-5, depending on the Si/Fe molar ratios of 20, 10, and 5, respectively.

**Figure 14:**
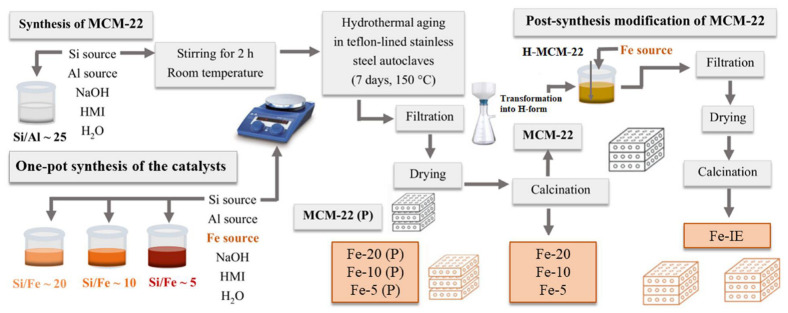
Schematic representation of the catalyst synthesis procedure.

The reference iron-modified MCM-22 was prepared by post-synthesis method, according to the procedure described for various zeolitic materials [41]. Typically, the pristine MCM-22 with Si/Al of ca. 25 was transformed into hydrogen form by ion-exchange with the aqueous solution of NH_4_NO_3_. Afterwards, the material was ion-exchanged with iron as it follows: 5 g of H-MCM-22 was kept in contact with the adequate volume of 0.05 mol dm^−3^ solution of Fe(NO_3_)_3_ 9H_2_O at 50 °C overnight. The resulting solid was filtered, washed several times with distilled water, and calcined at 550 °C for 6 h in air. The obtained sample was labeled as Fe-IE. The schematic presentation of the synthesis of the catalysts is demonstrated in Figure 14 and the codes of the prepared samples with the corresponding desired Si/Fe molar ratios are listed in Table 7.

### 4.2. Catalytic Activity Measurement

The catalytic performance of the prepared materials in NH_3_-SCR reaction was analyzed in a fixed-bed flow microreactor with a quartz tube, under atmospheric pressure. Typically, 200 mg of the catalyst with 20–35 mesh was outgassed in a flow of nitrogen at 400 °C for 30 min. The material was cooled down to 100 °C and exposed to the gas mixture containing 800 ppm of NO, 800 ppm of NH_3_, 3.5 vol.% of O_2_, and He as an inert. The total gas flow was 100 cm^3^ min^−1^. The NH_3_-SCR tests were carried out in a temperature range of 150 °C to 450 °C with 50 °C as a step. Concentration of the residual NO and N_2_O (the by-product of the reaction) in the outlet gas were analyzed continuously by the FT-IR detector (ABB 2000, AO series). The conversion of NO was calculated according to the formula represented by Equation (1):

(1) $$NO_{conv}=\frac{\left(C_{NO_{in}\;}-C_{NO_{out}}\right)}{C_{NO_{in}}}\;\cdot 100\%$$

where $C_{NO_{in}}$—inlet concentration of NO, $C_{NO_{out}}$—outlet concentration of NO in the gas mixture. 

In order to analyze the resistance of the catalysts to the poisoning with SO_2_, the materials were exposed to the gas mixture composed of 1000 ppm of SO_2_ in helium at 350 °C for 1 h. Directly after the poisoning mode, NH_3_-SCR catalytic tests were performed. The obtained conversion of NO after poisoning with SO_2_ was calculated by Equation (1).

### 4.3. Characterization of the Catalysts

The chemical composition of the catalysts in relation to the content of Si, Al, and Fe was determined by inductively coupled plasma optical mass spectroscopy (ICP-OES, QTEGRA). Crystallinity and phase purity were analyzed by X-ray powder diffraction (XRD). The XRD patterns were obtained using a Panalytical Empyrean diffractometer (Malvern Panalytical Ltd., Malvern, UK). The diffractograms were collected using Cu Kα radiation source (λ = 1.54184 Å) at a tube current of 40 mA and a voltage of 40 kV in the scanning range of *2θ* of 2–40° and 3–90°. The recorded XRD data were analyzed using X’pert HighScore plus software with database (Malvern Panalytical Ltd., Malvern, UK). The relative crystallinities (RC) of the catalysts were calculated according to the method presented by Xing and co-workers [95]. The sum of the intensities of diffraction maxima at *2θ* of 14.3°, 22.7°, 23.7°, and 26.0° of iron-modified samples were compared to that exhibited by MCM-22 (assumed as a reference with 100% crystallinity). Low-temperature N_2_ sorption measurements were conducted using Micromeritics 3Flex Surface Characterization gas adsorption analyzer (Micromeritics, Norcross, GA, USA). Prior to the analysis, the samples were degassed under vacuum at 90 °C for 1 h and afterwards, at 350 °C for 5 h. The specific surface area (*S_BET_*) of the materials was calculated using BET (Brunauer-Emmet-Teller) model, from the adsorption branch, according to the recommendations of Rouquerol [96]. Taking into consideration the shape of the isotherms, pointing at the micro-mesoporous structure of the catalysts, the external surface area, surface of micro- and mesopores, and their volume, were calculated using t-plot and BJH methods. Scanning electron microscopy (SEM) studies were carried out with the aid of Field Emission Scanning Electron Microscope JEOL JSM-7500F (JEOL, Tokyo, Japan) coupled with an AZtecLiveLite Xplore 30 (Oxfrod Instruments, Abingdon, UK) system. SEI and BSE (COMPO) images were provided by the secondary electron detector and back scattered electron detector, respectively. Transmission electron microscopy (TEM) was performed on a Tecnai TF20 X-TWIN (FEG) microscope (Thermo Fisher Scientific, Waltham, MA, USA) equipped with an EDS detector (EDAX), working at an accelerating voltage of 200 kV. Samples for TEM observations were prepared by drop-casting of powder/isopropyl alcohol suspensions on carbon coated copper TEM grids. The X-ray photoelectron spectroscopy (XPS) experiments were conducted in a PHI VersaProbeII Scanning XPS system (Physical Electronics, ULVAC-PHI, Chigasaki, Japan) using monochromatic Al Kα (1486.6 eV) X-rays focused to a 100 µm spot and scanned over the area of 400 µm × 400 µm. The photoelectron take-off angle was 45° and the pass energy in the analyzer was set to 117.50 eV (0.5 eV step) for survey scans and 46.95 eV (0.1 eV step) to obtain high energy resolution spectra for the C 1s, O 1s, Na 1s, Fe 2p, Al 2p and Si 2p regions. A dual beam charge compensation with 7 eV Ar^+^ ions and 1 eV electrons were used to maintain a constant sample surface potential, regardless of its conductivity. All of the XPS spectra were charge-referenced to the unfunctionalized, saturated carbon (C–C) C 1s peak at 284.8 eV. The operating pressure in the analytical chamber was less than 3 × 10^−9^ mbar. The spectra were deconvoluted using PHI MultiPak software (v.9.9.0.8) (ULVAC-PHI, Chigasaki, Japan). The spectrum background was subtracted by the Shirley method. Thermogravimetric studies (TG) were performed to determine changes in the weight of the catalyst within the temperature range of 30–800 °C, with the temperature ramp of 10 °C min^−1^. Typically, the sample was placed on a platinum holder, in the mixture of air and helium. The weight loss was investigated using Q5000 IR thermobalance (TA Instruments, Waters, New Castle, DE, USA). The concentration and strength of the acid sites was determined by temperature programmed desorption of NH_3_ (NH_3_-TPD) using Autochem II (Micrometrics, Norcross, GA, USA) apparatus. The experiments were performed in the temperature range of 100–800 °C in a fixed bed continuous flow microreactor. Prior to the measurement, each sample was pretreated in a stream of Ar at 100 °C for 60 min. Afterwards, the materials were equilibrated at 100 °C in a stream of He and saturated for 30 min in a flow of 1 vol.% of NH_3_ in He. Subsequently, the analyzed materials were heated with a temperature ramp of 10 °C min^−1^, up to 800 °C in Ar stream. The desorbed amount of ammonia was analyzed by the means of a thermal conductivity detector (TCD) and coupled GC-MS mass spectrometer (OmniSTAR, Leidschendam, The Netherlands). The NH_3_ uptake was calculated from the amount of desorbed gas volumes from the area under NH_3_-TPD curve. The setup was calibrated prior to the analysis with the known amounts of ammonia, to determine the precise area of a single pulse, registered by TCD detector. The characteristic chemical groups present in the zeolitic frameworks of MCM-22 and the catalysts were studied by FT-IR spectroscopy. The spectra were collected using Perkin Elmer Frontier spectrometer in the wavelength region of 4000–400 cm^−1^, with a resolution of 4 cm^−1^. The coordination and aggregation of iron species introduced into the zeolites were determined by ultraviolet diffuse reflectance spectra (UV-vis-DR). The analysis was carried out using Cary 5 spectrophotometer, equipped with a diffuse reflectance accessory. The spectra were taken in the range of 200–800 nm, with a resolution of 2 nm.

## 5. Conclusions

In this work, we intended to prepare Fe-MCM-22 with Si/Fe molar ratio of 20, 10, and 5 by one-pot synthesis method and test the materials in NH_3_-SCR reaction. In addition, we analyzed the resistance of the catalysts to the poisoning with sulfur dioxide. Catalytic performance and physicochemical properties of the materials were compared to the sample with Fe introduced by post-synthesis (ion-exchange) method. The results reported here confirmed that the addition of Fe precursor to the synthesis pot resulted in the introduction of Fe^3+^ into the zeolitic framework in a form of well-dispersed cations or deposition of extra framework iron species, depending on the concentration of Fe. We have observed that Si/Fe strongly influenced physicochemical parameters of the obtained materials. As a consequence, the catalysts exhibited various catalytic performances, determined mainly by the speciation of the active phase. We observed that Fe-20 exhibited the highest activity in NO conversion of ca. 100% at 175 °C and negligible N_2_O emission in the entire tested temperature range. The outstanding low-temperature performance of this sample was assigned to the abundance of isolated Fe^3+^ cations incorporated into the zeolitic framework and improved acidity, not detected for the post-synthesized material. Nevertheless, one should note that the sample was low in other types of iron sites, which resulted in slightly dropped, but quite constant NO conversion in high-temperature region of the reaction. On the other hand, the increase of iron loading contributed to the gradual reduction of the catalytic activity, which was ascribed to the formation of catalytically-passive and pore-clogging Fe_2_O_3_ bulks and restricted formation of microporous structure. All in all, we have proven that one-pot synthesis was beneficial to low-temperature activity, since NO conversion for the reference sample below 350 °C was significantly lower. Additionally, moderate concentration of the oligomeric Fe_x_O_y_ species in Fe-10 prevented the extensive formation of N_2_O during the reaction. Basing on the earlier reports, we ascribed this effect to the catalytic activity of those moieties in N_2_O decomposition. Interestingly, the results of our study supported the theory that not acidity, but iron loading and thus, its speciation and distribution were the key factors of catalytic performance in NH_3_-SCR. However, since the surface acidity decreased linearly with the increasing amount of the active phase, it can be assumed that bigger aggregates of metal oxide could block the acidic centers or plug the pores, hindering diffusion of the gas molecules through the structure of the materials. The results of catalytic tests after poisoning with SO_2_ indicated that ammonium sulfates and bisulfates lowered the activity of the materials below 250 °C. After decomposition of these salts, the activity was restored, suggesting reversible deactivation. What is more, *T_50_* of the catalysts moderately increased after the contact with SO_2_, however, high-temperature activity of the materials was almost completely preserved. Given these points, our findings provided an insight into the preparation of low-cost, non-toxic, ecological-friendly, highly active, and selective NH_3_-SCR catalysts, which can be synthesized by only one synthesis step.

## Figures and Tables

**Table 1:** N_2_O concentration (in ppm) at the outlet of the reactor, detected for the fresh and SO_2_-poisoned catalysts.

Temperature (°C)	Fe-20		Fe-10		Fe-5		Fe-IE	
	Fresh	SO_2_	Fresh	SO_2_	Fresh	SO_2_	Fresh	SO_2_
150	0	0	3	1	1	38	0	0
200	5	12	7	8	2	33	4	4
250	2	22	9	15	10	31	8	10
300	1	22	9	17	12	32	14	19
350	5	19	5	16	12	32	17	21
400	0	15	0	15	12	32	12	18
450	0	15	0	14	14	24	9	17

**Table 2:** Chemical composition and relative crystallinity of the samples.

Sample Code	Si (wt.%)	Al (wt.%)	Fe (wt.%)	Si/Al	RC (%)
MCM-22	33.23	1.41	0	23	100.0
Fe-20	36.38	1.17	4.78	29	65.5
Fe-10	36.58	1.60	8.43	21	15.8
Fe-5	33.32	1.40	14.90	23	0.0
Fe-IE	30.48	1.19	5.74	25	90.3

**Table 3:** Textural and structural properties of the samples.

Sample Code	Surface Area (m^2^ g^−1^)			Pore Volume (cm^3^ g^−1^)		
	S_BET_^a^	S_micro_^b^	S_Ext_^b^	V_total_	V_micro_^b^	V_meso_^c^
MCM-22	479	367	112	0.518	0.192	0.116
Fe-20	419	335	84	0.479	0.160	0.196
Fe-10	216	116	101	0.475	0.055	0.246
Fe-5	144	15	130	0.379	0.007	0.170
Fe-IE	372	263	109	0.418	0.129	0.119

^a^ Surface area determined by BET method. ^b^ Micropore surface area, external surface area, and micropore volume determined by t-plot method. ^c^ Average mesopore diameter and volume determined by BJH method.

**Table 4:** Weight loss of the samples determined by TGA experiments.

Sample Code	Weight Loss in the Temperature Range (%)		
	30–150 °C	150–400 °C	400–800 °C
MCM-22	4.3	0.6	0.6
Fe-20	7.5	1.4	0.7
Fe-10	3.3	0.9	0.6
Fe-5	2.1	0.9	0.5
Fe-IE	5.1	1.0	0.6

**Table 5:** The values of binding energy (eV) and relative areas of the components in atomic % of the core excitation of Si 2p, Al 2p, O 1s, and Fe 2p_3/2_ present in the catalysts.

Core Excitation	Binding Energy (eV)	Relative Area of the Component (at.%) in the Sample			
		Fe-20	Fe-10	Fe-5	Fe-IE
Si 2p	103.0	26.3	26.0	24.3	25.8
Al 2p	74.4	1.1	1.2	1.1	1.4
O 1s *	534.2–530.5	62.4	60.8	64.4	56.9
Fe 2p_3/2_ **	709.9	0.9	1.2	2.4	0.8

* Summary surface composition of O–Fe, O–Si, O=C, O–C, –OH, and H_2_O _(ads)_. ** The relative area of the component with the lowest binding energy

**Table 6:** Density of the acid centers in the samples determined by NH_3_-TPD.

Sample Code	Concentration of Acid Sites (μmol g^−1^)		
	Weak Sites	Medium and Strong Sites	Total Amount of Sites
MCM-22	747	524	1271
Fe-20	745	597	1342
Fe-10	398	219	617
Fe-5	153	0	153
Fe-IE	670	533	1203

**Table 7:** List of the prepared samples with their codes and corresponding calculated * Si/Fe molar ratios.

Sample Code	Si/Fe
MCM-22	non-modified support
Fe-20	20 *
Fe-10	10 *
Fe-5	5 *
Fe-IE	11 **

** Obtained Si/Fe molar ratio.

## Data Availability

Data are contained within the article.

## Supplementary Materials

The following supporting information can be downloaded at: https://www.mdpi.com/article/10.3390/ijms231810754/s1.

## Abbreviations

BET	Brunauer: Emmet, and Teller specific surface area
BJH	Barret-Joyner-Halenda pore size and volume
FT-IR	Fourier transform infrared spectroscopy
ICP-OES	Inductively couples plasma optical emission spectroscopy
MWW	Mobile Twenty Two type of framework
NH_3_-SCR	Selective catalytic reduction of nitrogen oxides with ammonia
NH_3_-TPD	Temperature-programmed desorption of ammonia
SEM/EDS	Scanning electron microscopy and electron-dispersive X-ray spectroscopy
Si/Al	Silicon to aluminum molar ratio
Si/Fe	Silicon to iron molar ratio
TEM BF	Bright-field transmission electron microscopy
TEM HAADF	High-angle annular dark-field scanning transmission electron microscopy
TEM HR	High resolution transmission electron microscopy
TGA	Thermogravimetric analysis
UV-Vis-DRS	Ultraviolet-Visible diffuse reflectance spectroscopy
XPS	X-ray photoelectron spectroscopy
XRD	X-ray diffraction
