# Supplementary material for: Catalytic Performance and Sulfur Dioxide Resistance of One-Pot Synthesized Fe-MCM-22 in Selective Catalytic Reduction of Nitrogen Oxides with Ammonia (NH3-SCR)—The Effect of Iron Content
Source: Int J Mol Sci. 2022 Sep 15;23(18):10754. doi: 10.3390/ijms231810754 (PMC9501588; doi:10.3390/ijms231810754)
Supplement: Supplementary file 1 [file ijms-23-10754-s001.zip › ijms-1906972-supplementary.pdf]

Catalytic Performance and Sulfur Dioxide Resistance of One-pot Synthesized Fe-MCM-22 in Selective Catalytic Reduction of Nitrogen Oxides with Ammonia (NH_3-SCR) – The Effect of Iron Content

Agnieszka Szymaszek-Wawryca [1,*], Urbano Díaz [2], Dorota Duraczyńska [3], Konrad Świerczek [1], Bogdan Samojeden [1], Monika Motak [1]

[1]*AGH University of Science and Technology, Faculty of Energy and Fuels*
[2] *Instituto de Tecnología Química, UPV-CSIC, Universidad Politécnica de Valencia, Avenida de los Naranjos, s/n 46022 Valencia, Spain*
[3]*Jerzy Haber Institute of Catalysis and Surface Chemistry, Polish Academy of Sciences, ul. Niezapominajek 8, 30-239 Kraków, Poland*
* Correspondence: agnszym@agh.edu.pl ; Tel.: +48 12 617 21 42

Supplementary Materials

The Electronic Supplementary Materials of the manuscript "Catalytic Performance and Sulfur Dioxide Resistance of One-pot Synthesized Fe-MCM-22 in Selective Catalytic Reduction of Nitrogen Oxides with Ammonia (NH_3-SCR) – The Effect of Iron Content" provides:

1. SEM images recorded for the investigated materials (Figure S1);
2. XPS spectra of the catalysts in BE region of O 1s (Figure S2);
3. XPS spectra of the catalysts in BE region of Si 2p (Figure S3);
4. XPS spectra of the catalysts in BE region of Al 2p (Figure S4);
5. XPS spectra of the catalysts in BE region of C 1s (Figure S5);
6. Deconvolution of the NH_3-TPD profile of the pristine MCM-22 (Figure S6);
7. Deconvolution of the NH_3-TPD profile of Fe-20 (Figure S7);
8. Deconvolution of the NH_3-TPD profile of Fe-10 (Figure S8);
9. Deconvolution of the NH_3-TPD profile of Fe-5 (Figure S9);
10. Deconvolution of the NH_3-TPD profile of Fe-IE (Figure S10).

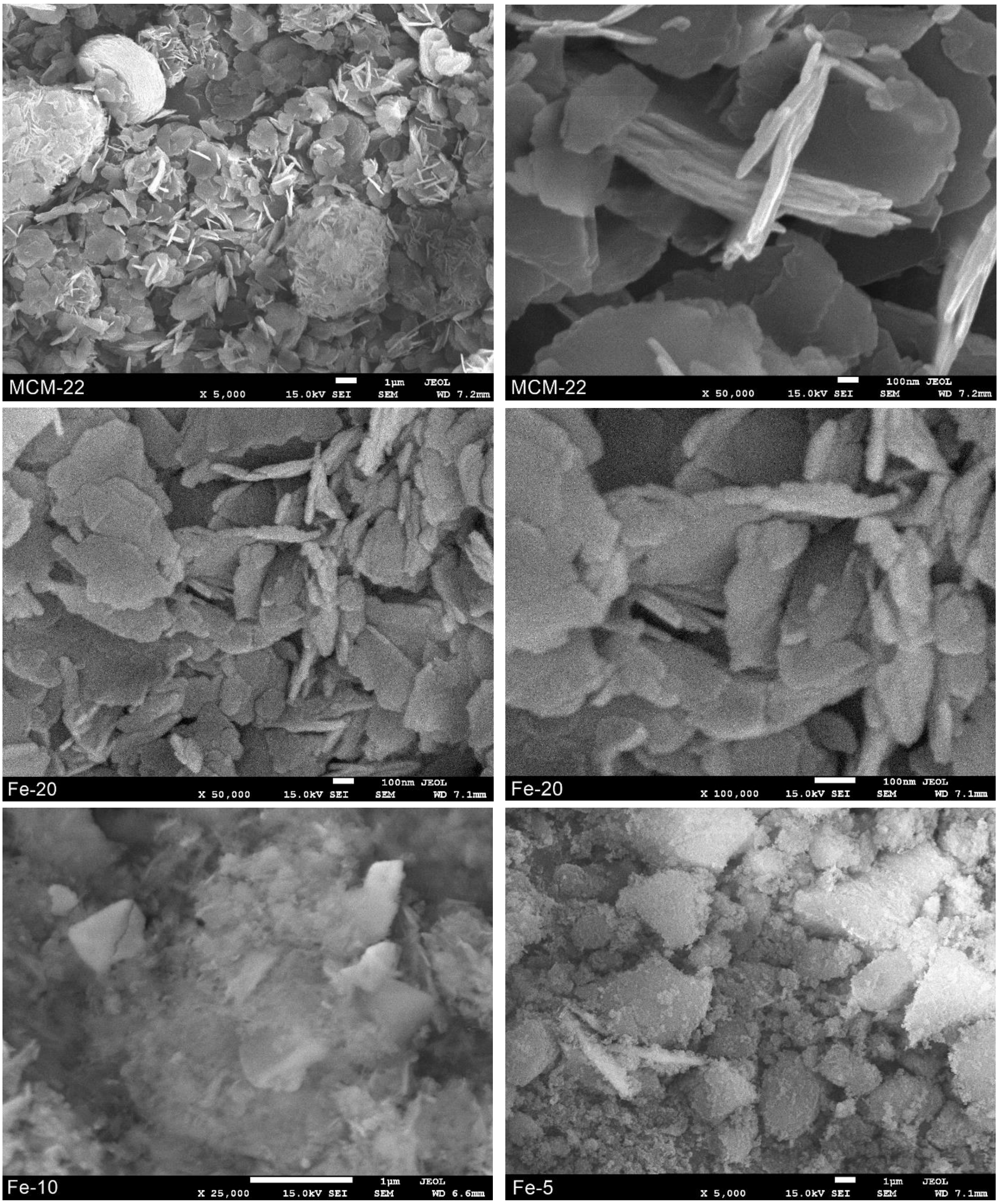
MCM-22
X 5,000 15.0kV SEI 1µm JEOL SEM WD 7.2mm
MCM-22
X 50,000 15.0kV SEI 100nm JEOL SEM WD 7.2mm
Fe-20
X 50,000 15.0kV SEI 100nm JEOL SEM WD 7.1mm
Fe-20
X 100,000 15.0kV SEI 100nm JEOL SEM WD 7.1mm
Fe-10
X 25,000 15.0kV SEI 1µm JEOL SEM WD 6.6mm
Fe-5
X 5,000 15.0kV SEI 1µm JEOL SEM WD 7.1mm

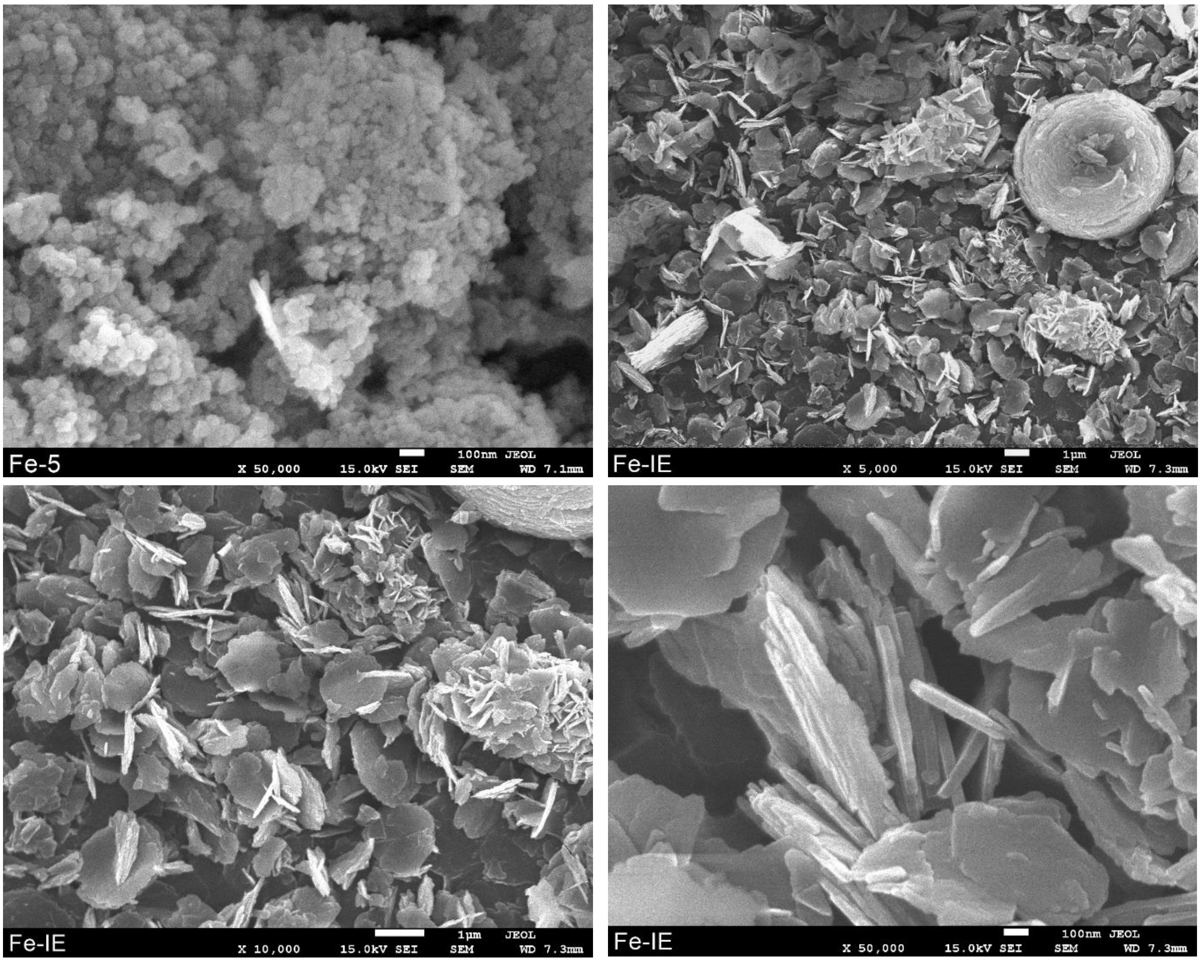

Figure S1. SEM images recorded for the investigated materials.

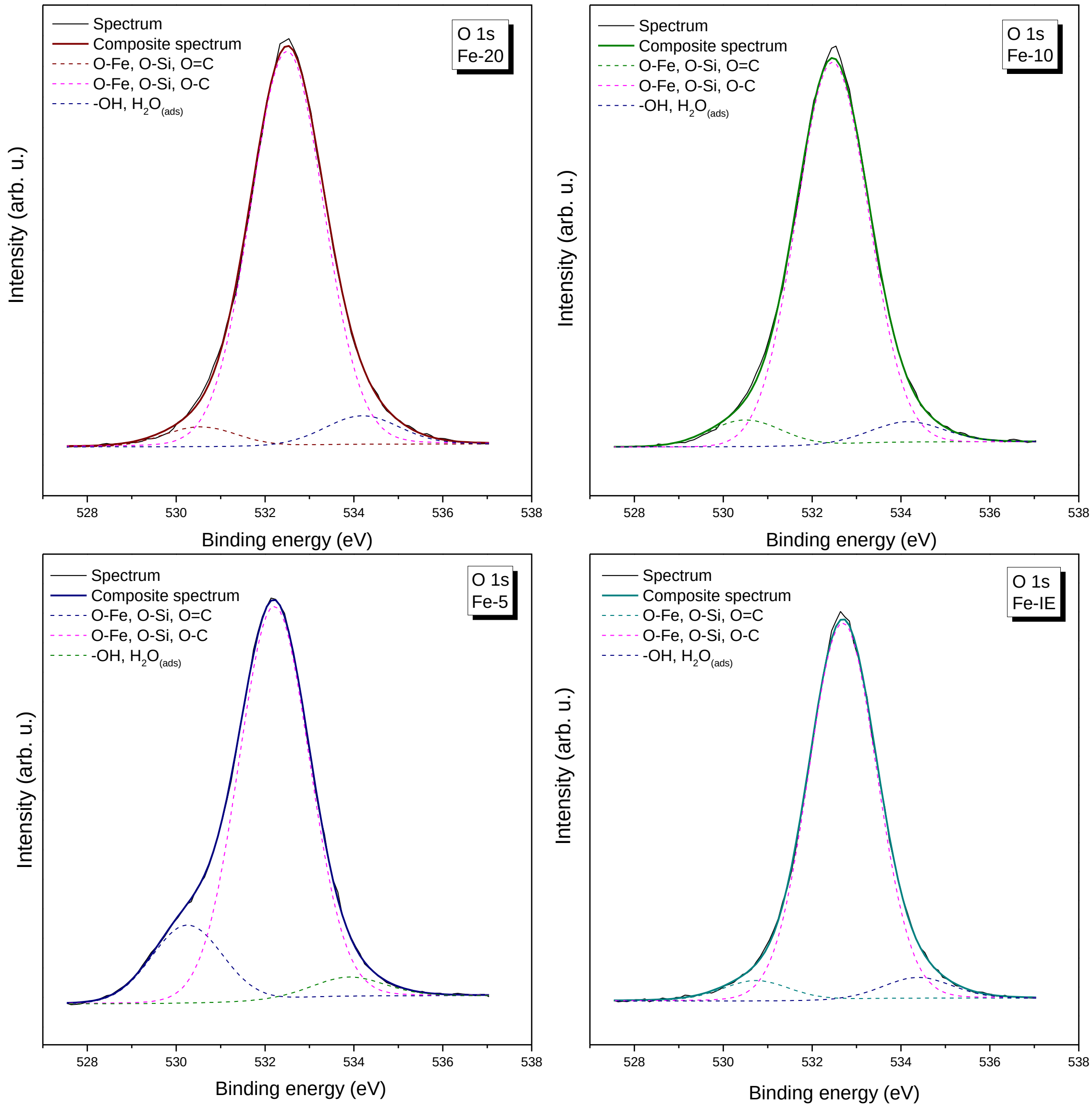

Figure S2. XPS spectra of the catalysts in BE region of O 1s.

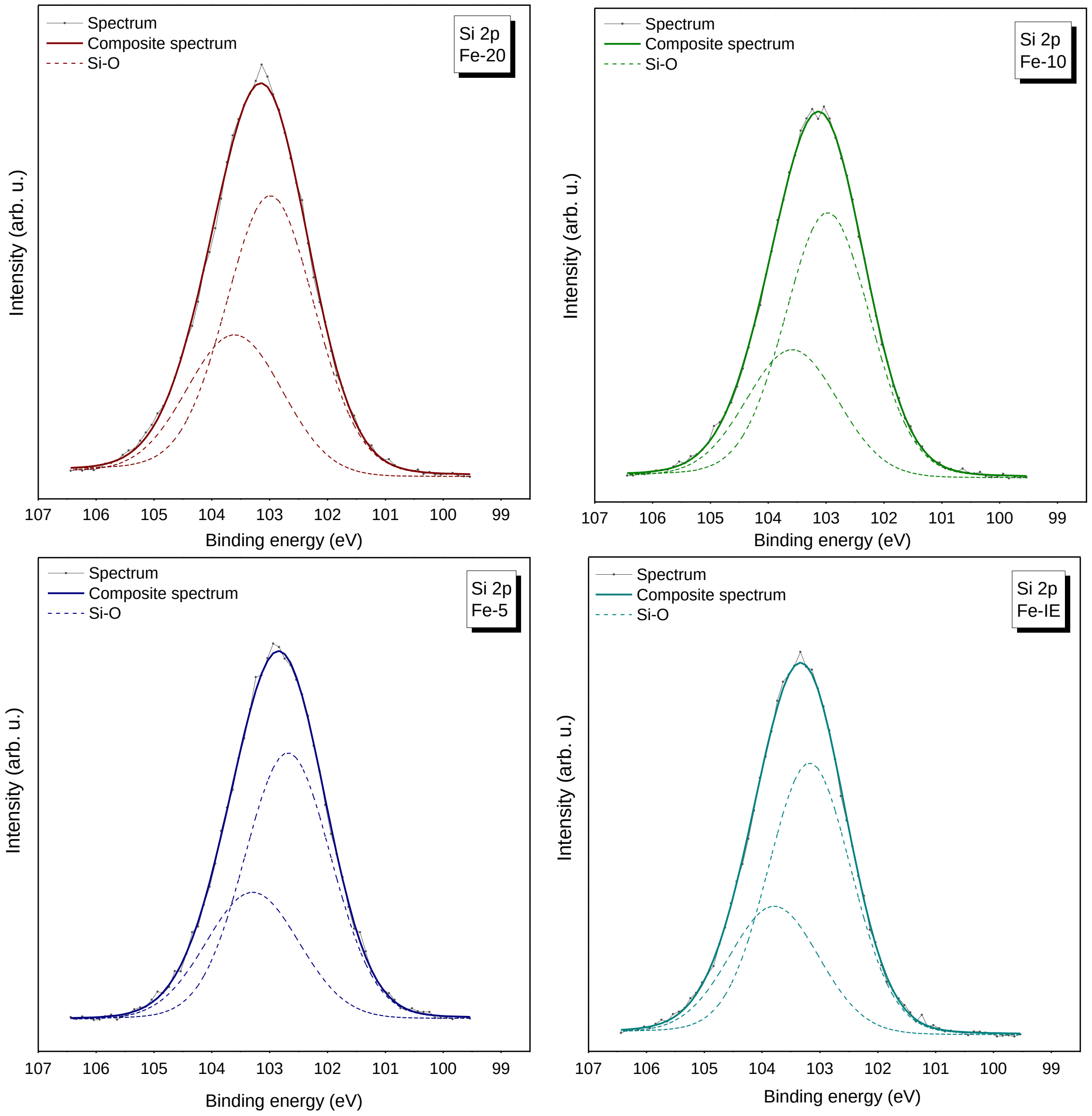

Figure S3. XPS spectra of the catalysts in BE region of Si 2p.

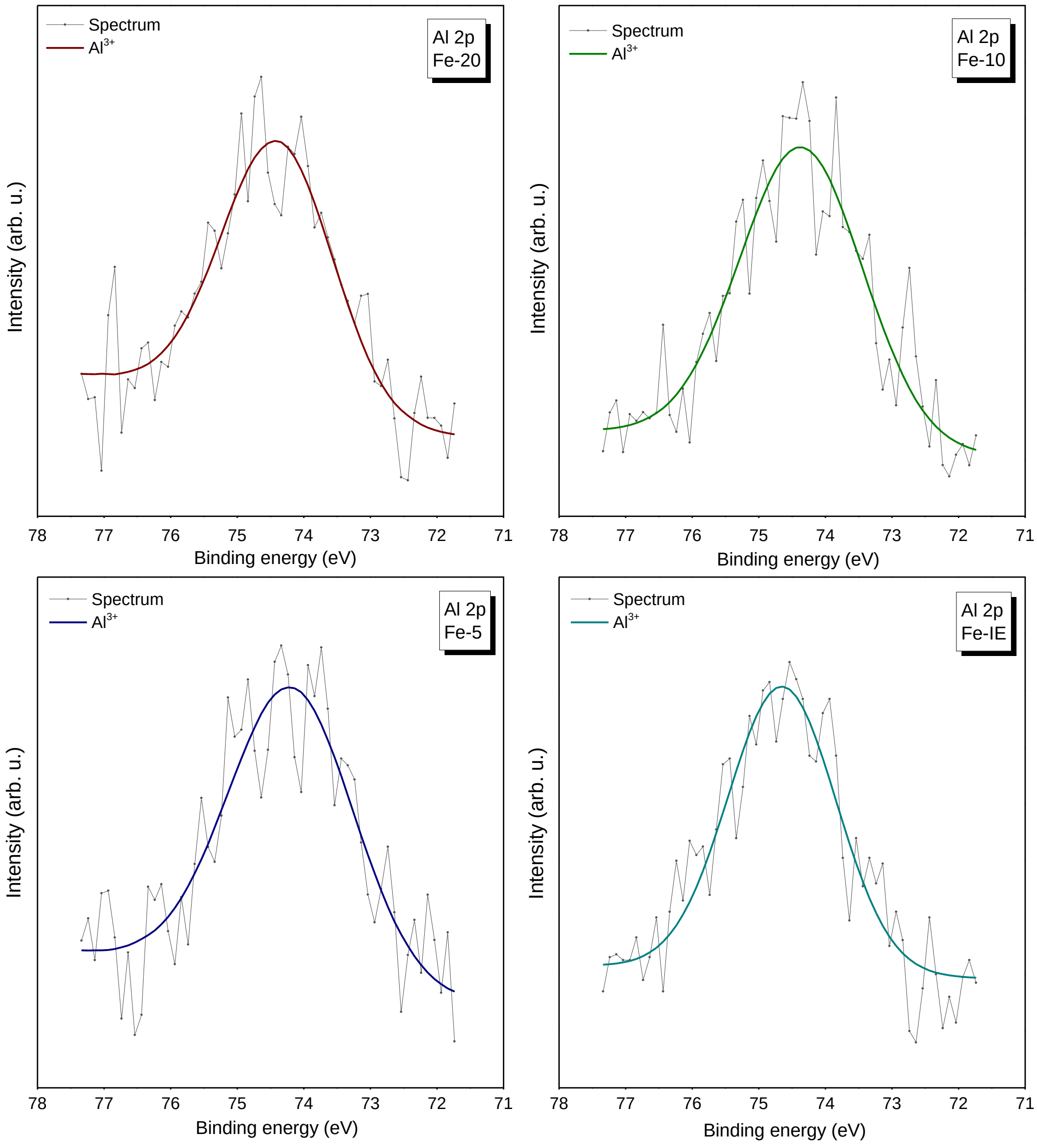

Figure S4. XPS spectra of the catalysts in BE region of Al 2p.

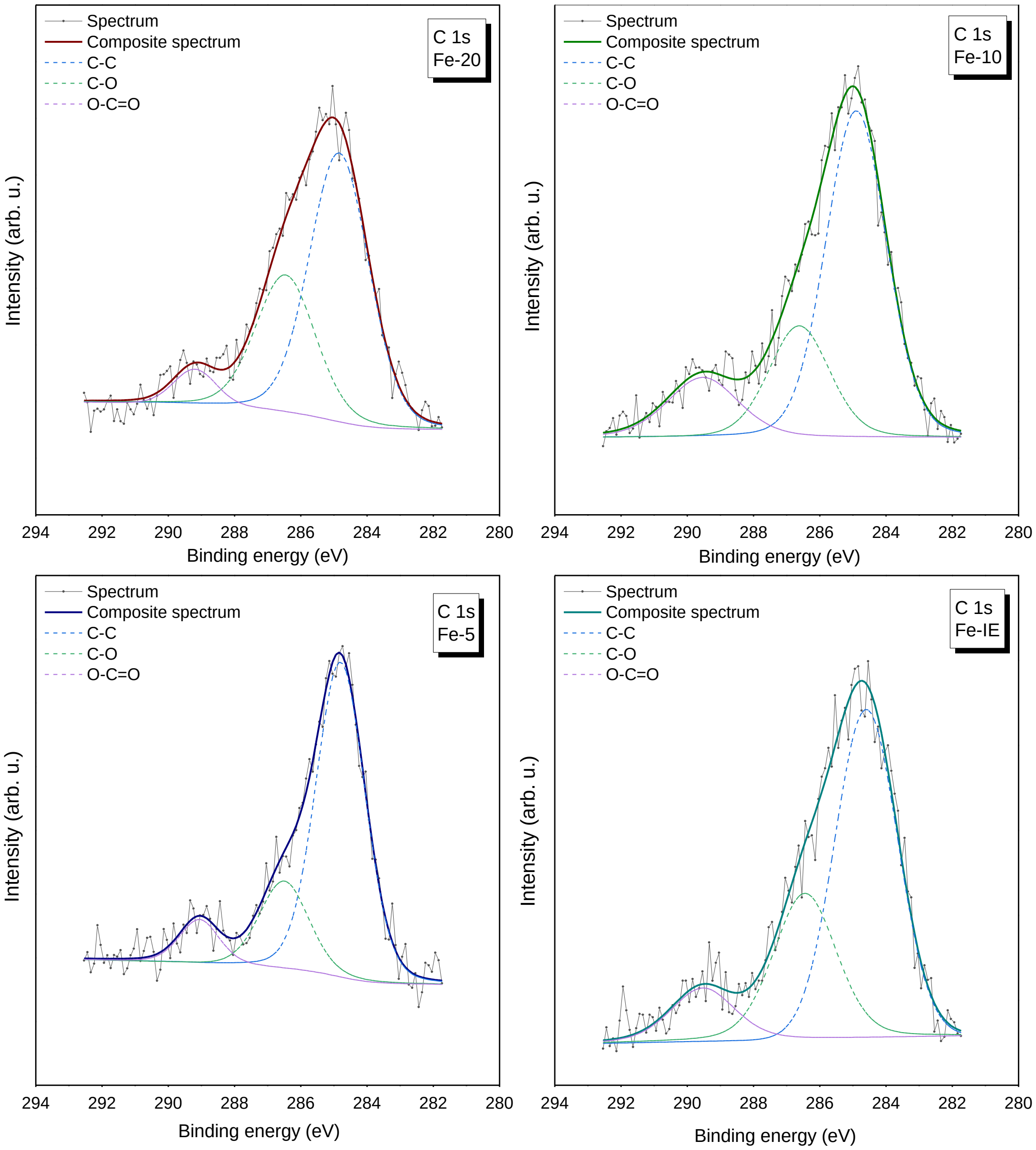

Figure S5. XPS spectra of the catalysts in BE region of C 1s.

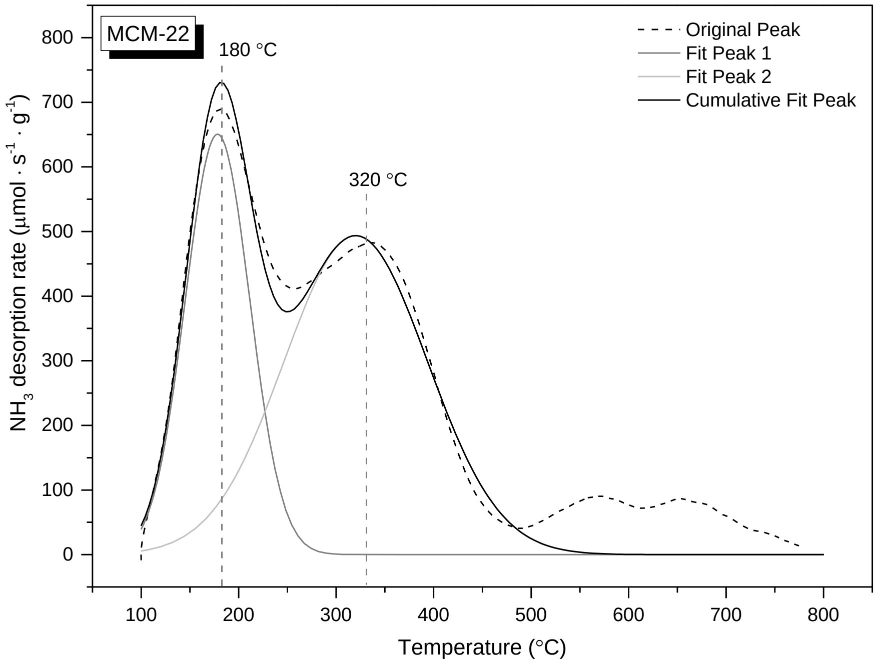

Figure S6. Deconvolution of the NH_3-TPD profile of the pristine MCM-22 (the profile was deconvoluted using Gaussian function).

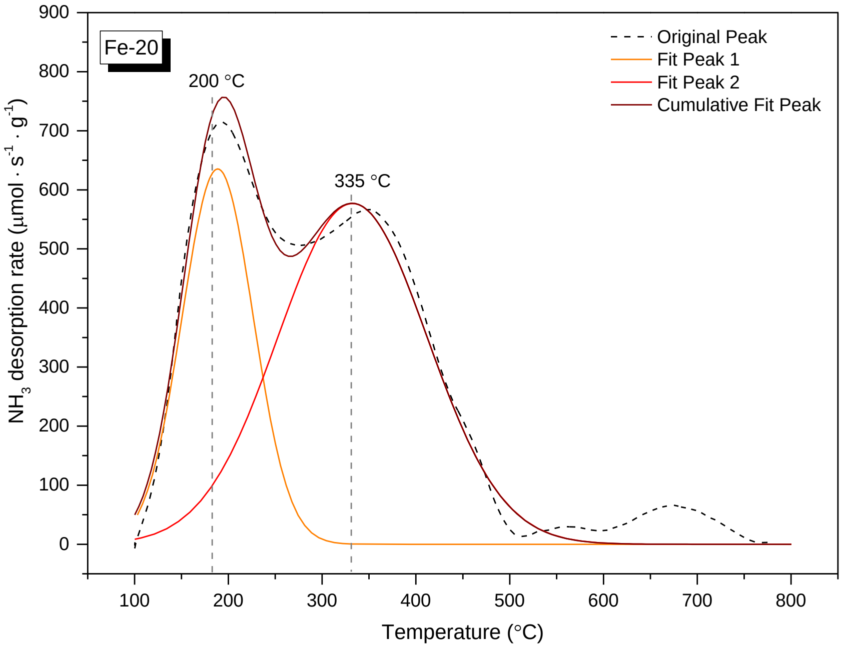

Figure S7. Deconvolution of the NH_3-TPD profile of the Fe-20 (the profile was deconvoluted using Gaussian function).

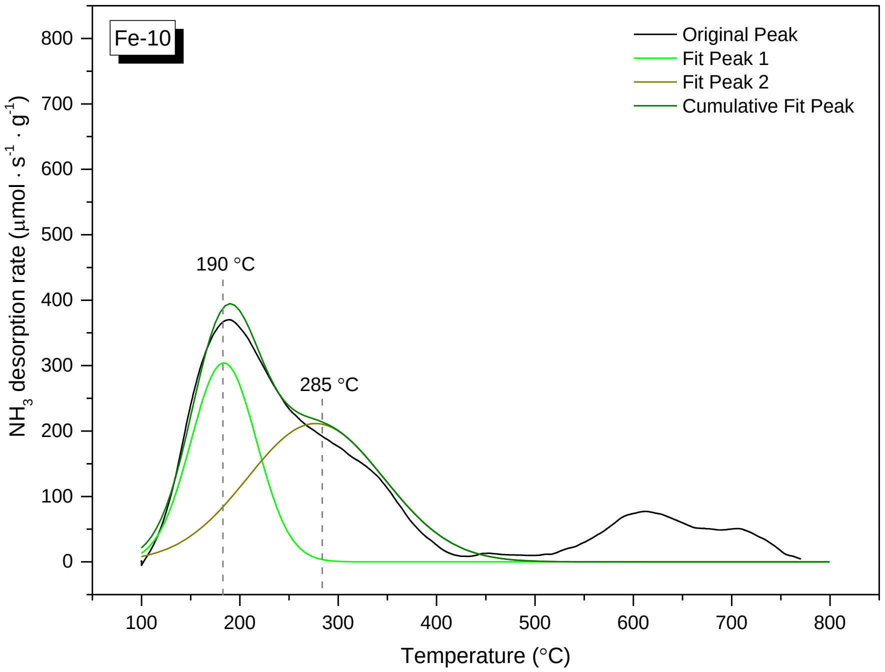

Figure S8. Deconvolution of the NH3-TPD profile of the Fe-10 (the profile was deconvoluted using Gaussian function).

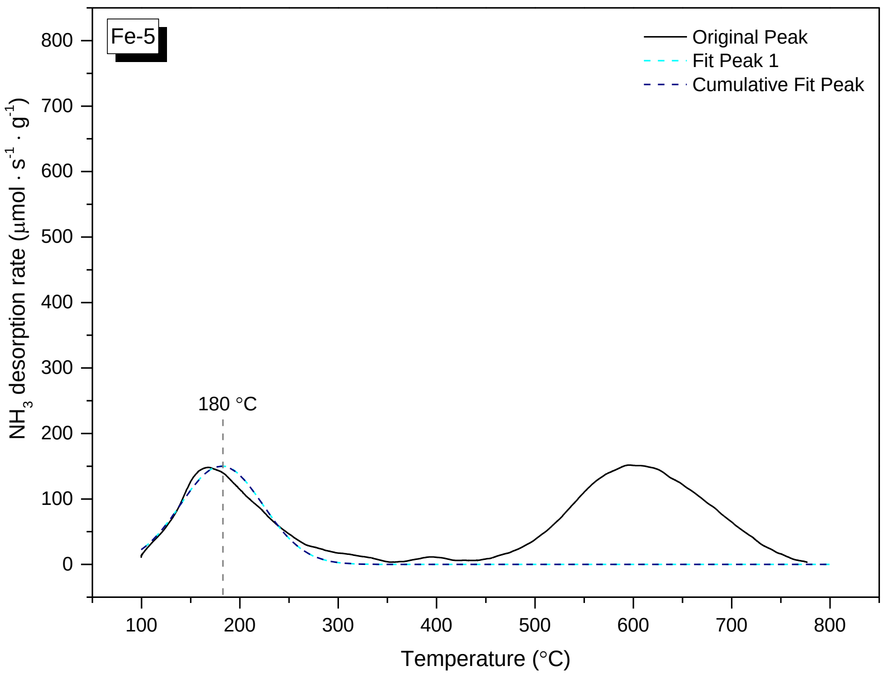

Figure S9. Deconvolution of the NH3-TPD profile of the Fe-5 (the profile was deconvoluted using Gaussian function).

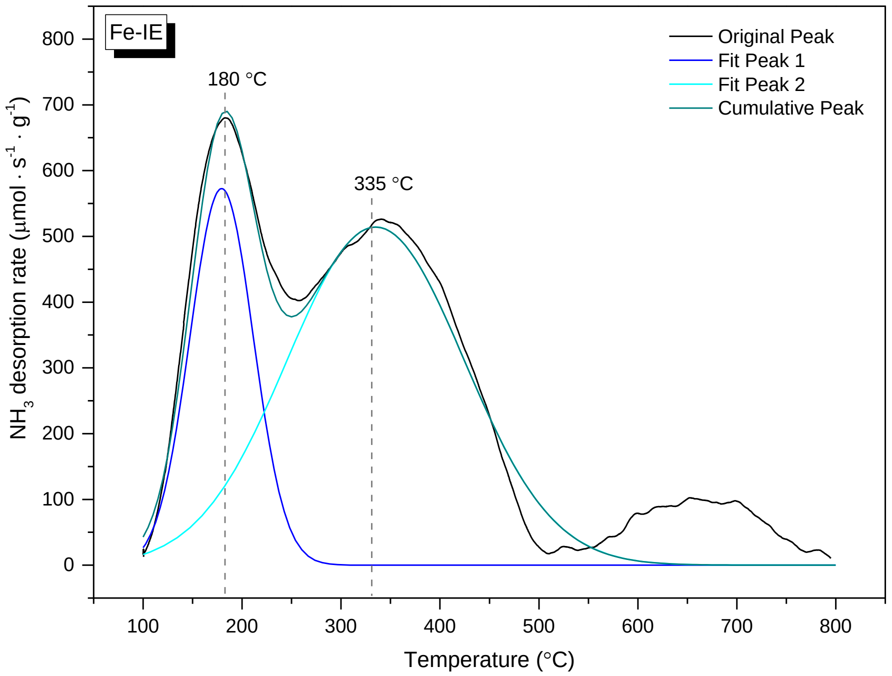

Figure S10. Deconvolution of the NH_3-TPD profile of the Fe-IE (the profile was deconvoluted using Gaussian function).
